# Mouse closed head traumatic brain injury replicates the histological tau pathology pattern of human disease: characterization of a novel model and systematic review of the literature

**DOI:** 10.1186/s40478-021-01220-8

**Published:** 2021-06-29

**Authors:** Aydan Kahriman, James Bouley, Thomas W. Smith, Daryl A. Bosco, Amanda L. Woerman, Nils Henninger

**Affiliations:** 1 Department of Neurology, Medical School, University of Massachusetts, 55 Lake Ave, Worcester, USA; 2 Department of Pathology, Medical School, University of Massachusetts, 55 Lake Ave, Worcester, USA; 3 Department of Biology, University of Massachusetts Amherst, Amherst, MA 01003 USA; 4 Department of Psychiatry, Medical School, University of Massachusetts, 55 Lake Ave, Worcester, USA

**Keywords:** Animal model, Chronic traumatic encephalopathy, Concussion, Systematic review, Tauopathy, Traumatic brain injury

## Abstract

Traumatic brain injury (TBI) constitutes one of the strongest environmental risk factors for several progressive neurodegenerative disorders of cognitive impairment and dementia that are characterized by the pathological accumulation of hyperphosphorylated tau (p-Tau). It has been questioned whether mouse closed-head TBI models can replicate human TBI-associated tauopathy. We conducted longitudinal histopathological characterization of a mouse closed head TBI model, with a focus on pathological features reported in human TBI-associated tauopathy. Male C57BL/6 J mice were subjected to once daily TBI for 5 consecutive days using a weight drop paradigm. Histological analyses (AT8, TDP-43, pTDP-43, NeuN, GFAP, Iba-1, MBP, SMI-312, Prussian blue, IgG, βAPP, alpha-synuclein) were conducted at 1 week, 4 weeks, and 24 weeks after rTBI and compared to sham operated controls. We conducted a systematic review of the literature for mouse models of closed-head injury focusing on studies referencing tau protein assessment. At 1-week post rTBI, p-Tau accumulation was restricted to the corpus callosum and perivascular spaces adjacent to the superior longitudinal fissure. Progressive p-Tau accumulation was observed in the superficial layers of the cerebral cortex, as well as in mammillary bodies and cortical perivascular, subpial, and periventricular locations at 4 to 24 weeks after rTBI. Associated cortical histopathologies included microvascular injury, neuroaxonal rarefaction, astroglial and microglial activation, and cytoplasmatic localization of TDP-43 and pTDP-43. In our systematic review, less than 1% of mouse studies (25/3756) reported p-Tau using immunostaining, of which only 3 (0.08%) reported perivascular p-Tau, which is considered a defining feature of chronic traumatic encephalopathy. Commonly reported associated pathologies included neuronal loss (23%), axonal loss (43%), microglial activation and astrogliosis (50%, each), and beta amyloid deposition (29%). Our novel model, supported by systematic review of the literature, indicates progressive tau pathology after closed head murine TBI, highlighting the suitability of mouse models to replicate pertinent human histopathology.

**Supplementary Information:**

The online version contains supplementary material available at 10.1186/s40478-021-01220-8.

## Introduction

Traumatic brain injury (TBI) represents a major public health problem affecting more than 10 million people worldwide each year [36]. TBI is a leading cause of adult death and disability worldwide. It has been estimated that annually 150 to 200/1,000,000 people become disabled as a result of brain trauma, and TBI was declared a major public health problem by the National Institutes of Health in 1999 [4, 20, 53].

There is a long history of epidemiological evidence that TBI represents one of the strongest environmental risk factors for several progressive neurodegenerative disorders of cognitive impairment and dementia that are characterized by the pathological accumulation of hyperphosphorylated tau (p-Tau). In particular, TBI has been linked to Alzheimer’s disease (AD) and a unique clinicopathological entity termed chronic traumatic encephalopathy (CTE) [8, 17, 29, 46, 66, 67]. Yet, the exact mechanism(s) driving pathological tau accumulation and spread, cognitive impairment, and dementia after TBI are poorly understood. Defining the mechanisms that explain the link between TBI and dementia at the cellular level is a public health priority [61].

Mouse models of TBI-associated pathology are of great value because of the ability to conduct detailed, longitudinal histopathological characterization in a temporally accelerated fashion, as well as the unique possibility to explore underlying molecular mechanisms through genetic manipulation without confounding by comorbid conditions. This appears particularly important in light of a current lack of a uniformly accepted definition for TBI-specific tauopathy. Although pathological consensus criteria have been developed for CTE [8, 46], which is considered the prototypical TBI-associated tauopathy, challenges in applying these criteria relate to the fact that many pathologies are also present in the normally aging brain and other neurodegenerative conditions [27, 38]. Hence, distinguishing the direct effects of TBI from a sporadic progressive neurodegenerative process in humans is difficult. Moreover, animal models are needed to dissect the specific neuropathology of TBI and how pathological tau deposition relates to progressive neurodegeneration. However, concerns have been raised that murine closed head injury models may not be suitable to replicate pertinent histopathological features of human TBI-associated tauopathy [18]. Depending on the model used, variability in post-TBI histopathology may be considerable [9, 10]. Further, there are distinct differences in the cerebral anatomy as well as expression of tau isoforms in the adult brains of mice and humans [32], which could conceivably contribute to disparate pathology [41]. Therefore, it is critical to understand to what degree murine closed-head TBI can replicate human TBI-associated tau pathology and related histopathological features.

In this study, we characterized the histopathological features of a mouse closed head repetitive TBI (rTBI) model with specific focus on the presence and evolution of p-Tau accumulation and its association with pertinent histopathological features of human TBI-associated tauopathy according to reported consensus criteria for human CTE [8, 46]. In addition, we conducted a systematic literature review of reported tau pathology in mouse closed-head TBI and its relationship to pertinent histological features of human CTE to provide a contemporary overview of the state of the field.

## Material and methods

### Mouse study

Mice were randomly allocated to sham surgery versus rTBI except for 4 mice allowed to survive for 24-weeks after rTBI, which were added to gain insight into the longer-term evolution of p-Tau and associated pathology. Histological analyses were conducted by an investigator masked to the experimental groups.

### Animals

Male C57BL/6 J mice (Jackson Laboratories) were socially housed with same-sex mice (n = 4 per cage) on 12-h light/dark cycle with food and water ad libitum. Spontaneously breathing mice (n = 27) weighing 26.8 ± 2.6 g (age 8–12 weeks) were subjected to closed head injury (n = 20) or sham surgery (n = 11). Brains were removed 1 week after rTBI (n = 8), 4 weeks (sham n = 8, rTBI n = 8), and 24 weeks (rTBI n = 4) for histological analyses. Additionally, we conducted AT8-staining in 3 sham operated mice that survived for 12 months to rule out any potential age-related effects on p-Tau pathology.

### Anesthesia, analgesia, and traumatic brain injury induction

Animals were anesthetized with isoflurane (5% for induction, 2% for surgery, 1.5% for maintenance) in room air. Anesthesia was discontinued immediately prior to TBI and sham injury. Body temperature was monitored continuously with a rectal probe and maintained at 37.0 ± 0.5 °C. To alleviate pain, animals received 0.05 mg/kg subcutaneous buprenorphine (Med-Vet International, Mettawa, Il, USA) 30 min before anesthesia and every 6 h afterwards for 24 h. Additionally, each animal received 5 mg/kg subcutaneous carprofen (Patterson Veterinary, Devens, MA, USA) at the end of the anesthesia.

TBI was produced using a weight drop device as previously described in detail [11, 31] with the modification that animals were subjected to TBI or sham injury once daily for 5 consecutive days. Briefly, a 50 g weight was freely dropped 15 cm to strike a cylindrical polyacetal transducer rod (Delrin^©^, tip-diameter 2 mm, 17.4 g) that was placed with its tip directly on the exposed skull (target 2.5 mm posterior and 2.5 mm lateral from Bregma). Following TBI, the wound closed with interrupted sutures. Sham animals were anesthetized, surgically prepared (including skin incision), and placed under the impact device with the impactor touching the skull, but were not subjected to head impact. One mouse with a skull fracture was excluded.

### Immunohistochemistry staining

For histology, animals received an overdose of pentobarbital (150 mg/kg Fatal-Plus, Vortech Pharmaceuticals). Then animals were perfused under isoflurane anesthesia through the ascending aorta with 50 mL saline and then with ice cold phosphate-buffered 4% paraformaldehyde (PFA) for 10 min. Brains were removed from the cranium, postfixed overnight in the same fixative, and then stored in 0.4% PFA at 4 °C until further processing. Prior to paraffin embedding brains were pre-sectioned using a brain matrix.

Immunohistochemistry was performed against p-Tau^Ser−202/Thr205^, (AT8, 1:250, Thermo Fisher Scientific, Cat# MN1020, RRID: AB_223647), p-Tau^Thr231^ (AT180, 1:250, Thermo Fisher Scientific, Cat# MN1040, RRID: AB_223649), TAR DNA-binding protein 43 (TDP-43, 1:250, Proteintech, Cat# 10,782–2-AP, RRID: AB_615042), pTDP-43^Ser−409/410^ (1:250, Proteintech, Cat# 22,309–1-AP, RRID: AB_11182943), neuronal nuclei (NeuN, 1:200, Proteintech, Cat# 26,975–1-AP, RRID: AB_2880708), glial fibrillary acidic protein (GFAP, 1:250, Agilent, Cat# Z0334, RRID: AB_10013382), ionized calcium binding adaptor molecule 1 (1:250, Iba-1, Wako, Cat# 019–19,741, RRID: AB_839504), myelin basic protein (MBP, 1:200, Santa Cruz Biotechnology, Cat# M3821, RRID: AB_1841021), SMI-312 (1:200, BioLegend Cat# 837,904, RRID: AB_2566782), beta amyloid precursor protein (βAPP, 1:200, Zymed, CT695, Cat# 51–2700, RRID: AB_2533902), α-synuclein (1:250, Biolegend, Cat# 824,301, RRID: AB_2564879), and immunoglobulin G (IgG, 1:100, Abcam, Cat# ab6708, RRID: AB_956005). For chromogenic staining, tissue sections labeled with the primary antibodies (AT8, AT180, NeuN, βAPP, α-synuclein, IgG) were incubated with appropriate biotin-conjugated secondary antibodies followed by avidin–biotin complex (Vector Laboratories) incubation and treatment with diaminobenzidine as directed by the manufacturer. Hematoxylin counterstaining was used for AT8, AT180, βAPP, and α-synuclein labeled tissues. For immunofluorescence staining tissue sections labeled with the primary antibodies (TDP-43, pTDP-43, NeuN, GFAP, Iba-1, MBP, SMI-312, βAPP, alpha-synuclein) were incubated in appropriate secondary antibodies conjugated with Alexa Fluor 488 (1:250, Abcam, Cat# ab150113, RRID: AB_2576208 and Cat# ab150077, RRID: AB_2630356), Alexa Fluor 555 (1:250, Abcam, Cat# ab150106, RRID: AB_2857373), and Alexa Fluor 647 (1:250, Abcam, Cat# ab150075, RRID: AB_2752244 and Cat# ab150115, RRID: AB_2687948). Omitting the primary antibody in a subset of slides served as negative controls.

### Prussian blue staining

To assess for microhemorrhages, sections were stained for Prussian blue reaction using an Iron Stain Kit (# HT20, Sigma-Aldrich), following the manufacturer’s instructions.

### Microscopy

**Fig. 1:**
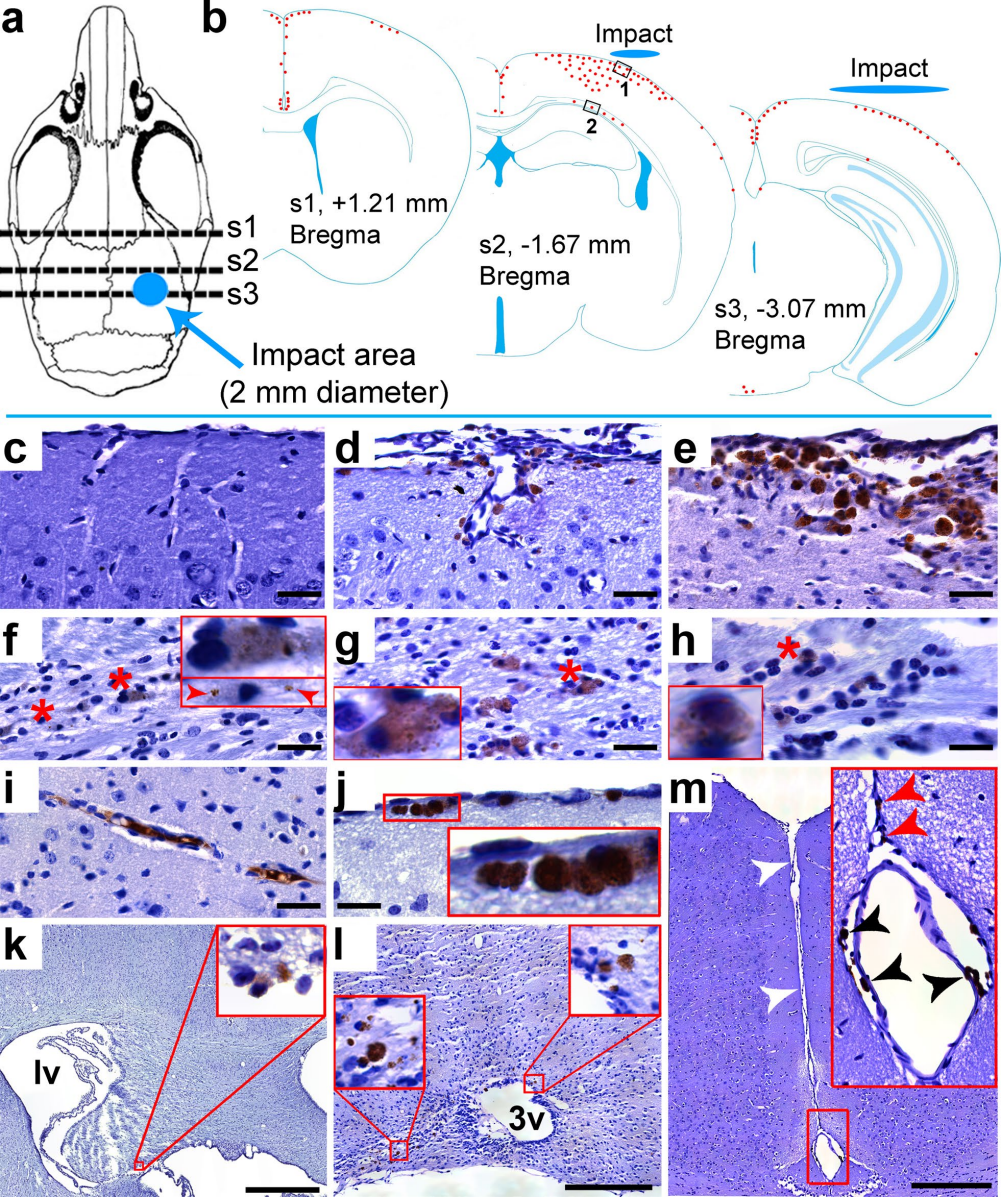
Patterns and evolution of p-Tau accumulation after closed head repetitive traumatic brain injury (rTBI). **(a)** Approximate location of the impact center over the intact mouse skull (blue circle) relative to the brain sections sampled for histological analysis (s1-s3; dashed lines). (**b**) Approximate location of p-Tau positive cells at 4 weeks after rTBI (composite of 8 mice; each red dot represents 8 p-Tau positive cells, blue ellipses indicate the spatial relation between the impactor and brain surface). Black boxes indicate the location of photomicrographs shown in panel c-e (box 1) and f–h (box 2). (**c**) Intact cerebral cortex without p-Tau accumulation at 1 week after rTBI. Progressive accumulation of p-Tau in the superficial layers of the cerebral cortex at (**d**) 4 weeks and (**e**) 24 weeks after rTBI. P-Tau accumulation in the corpus callosum at (**f**) 1 week, (**g**) 4 weeks, and (**h**) 24 weeks after rTBI (red asterisks). (f) In contrast to later time points, AT8-immunoreactivity at 1 week was restricted to dot-like staining in a subset of cells (arrowheads). Examples of p-Tau accumulation in (**i**) perivascular, (**j**) subpial, (**k, l**) periventricular, and (**l**) mammillary body locations as well as (**m**) at the depth of the superficial longitudinal fissure (white arrowheads) in perivascular (black arrowheads) and subpial (red arrowheads) locations at 4 weeks after rTBI. Scale bars are 30 µm (in **c–j**), 1 mm (**k**), and 300 µm (in **l–m**)

Paraffin sections, 10-µm thick coronal, were obtained at approximately Bregma -3.07 mm (impact center; s3), −1.67 mm (adjacent to the impact center; s2), and + 1.21 mm (remote to the impact center; s1) for histological assessment (Fig. 1a, b). For quantitative and qualitative analyses of AT8, NeuN, GFAP, Iba-1, MBP, and SMI-312 staining one coronal section each from these coordinates (s1-s3) was used. For qualitative analyses of βAPP, alpha-synuclein, IgG, and Prussian blue staining we reviewed one coronal section each from s1-s3. For quantitative and qualitative analyses of TDP-43 and pTDP-43 one coronal section from s2 was used. All histological analyses were performed by an investigator masked to the animal groups.

### Image acquisition and quantification

To acquire images of all stained sections for subsequent offline analysis we used a Leica DM6 B microscopy system equipped with a brightfield DMC5400 color CMOS camera and an immunofluorescent DFC9000 sCMOS camera.

To determine the spatial distribution of p-Tau accumulation after rTBI, sections were imaged at 63 × magnification and the approximate location of all AT8-positive profiles systematically recorded within each of the sampled sections and transferred to a standard atlas [52] to provide a visual representation of the observed tau pathology relative to the impact location. Given reported p-Tau accumulation in both neurons and astrocytes in human and mouse TBI, we quantified the number of p-Tau positive cells across time points as stratified by neuronal versus astrocytic p-Tau. Moreover, because we observed the earliest p-Tau accumulation in the corpus callosum with only later involvement of the cerebral cortex we additionally stratified these analyses according to the location in the superficial cortex (approximately cortical layers I-III), deep cortex (approximately cortical layers IV-VI), and the corpus callosum.

To determine the extent of neuronal loss, chromogen stained NeuN-positive cells were assessed in each coronal section. Images of 16 nonoverlapping fields of view (FOV; 8 per hemisphere; 659 × 439 µm, each) covering the dorsal cerebral cortex [11] were taken at 20 × magnification. NeuN positive cells were semiautomatically quantified using ImageJ [60] as previously described [15]. First, 16-bit color images were converted to 8-bit grayscale followed by automatic thresholding. The Analyze Particle tool was then used to count all particles with a circularity of 0.1–1.0 and a size of 25 to 250 µm^2^. Results were adjusted by manually counting all overlapping cells (ie, particle size greater than 250 µm^2^).

To assess microglia and astroglia in the dorsal cortex we used fluorescence staining. For GFAP, images of 16 nonoverlapping FOVs (8 per hemisphere; 667 × 667 µm, each) covering the dorsal cerebral cortex were taken at 20 × magnification. First, 16-bit color images were split into the individual color channels (red, green, blue) followed by automatic thresholding of the 8-bit green channel and black-white color inversion. The Analyze Particle tool was then used to quantify the total thresholded area (µm^2^). For Iba-1, TDP-43, and pTDP-43 images of 16 FOVs (8 per hemisphere; 211 × 211 µm, each) centered within the corresponding FOV used for the GFAP analyses were taken at 63 × magnification and analyzed as described for GFAP.

To assess the impact of rTBI on axonal integrity in the injured cortex, we used fluorescence staining for the myelin marker MBP and pan-axonal neurofilament marker SMI312. Images of one FOV (206 × 206 µm) per section were taken at 63 × magnification and analyzed as described for GFAP.

### Assessment of chronic traumatic encephalopathy (CTE) related pathology

With respect to assessing CTE-like pathology in our model, we defined histopathological features according to recently published consensus group criteria developed by an NINDS/NIBIB panel [8, 46] with modifications for use in mice (Table 1). Specifically, a defining criterion for human CTE includes the presence of perivascular foci of p-Tau immunoreactive neurofibrillary tangles (NFTs) and abnormal neurites, with or without p-Tau immunoreactive astrocytes, in an irregular pattern in the cerebral cortex, with a tendency to involve the sulcal depths. Because the lissencephalic brains of mice lack sulci, we considered the presence of perivascular p-Tau in the cerebral cortex a pathognomonic criterion. With respect to supportive features, we assessed for several supportive tau- and nontau-related histopathological features (Table 1). We did not assess for macroscopic pathology such as dilation of ventricles, septal pathology, atrophy, contusions, or other signs of previous trauma [8, 46]. Finally, for additional context we also assessed several pathological features that have been repeatedly described in human TBI but are not specific to CTE and may be shared with TBI and other neurodegenerative conditions including the presence of reactive microglia, astrogliosis, neuronal and axonal loss, presence of beta amyloid and alpha-synuclein depositions as well as evidence of prior blood brain barrier (BBB) disruption and microvascular injury/cerebral microbleeds [45, 49].

**Table 1:** Chronic traumatic encephalopathy (CTE) related pathology as adapted from consensus criteria [8, 46] and including frequently associated pathology [38, 45, 48, 49] for use in mice in the systematic review and characterization of our mouse model

	1 week	4 weeks	24 weeks
*Neuropathology considered Pathognomonic for CTE*			
Perivascular p-Tau accumulation			
P1: p-Tau immunoreactive neurons	–	x	x
P2: p-Tau immunoreactive astrocytes	–	x	x
*Neuropathological features Supportive of CTE*			
P-Tau related pathologies			
S1: Cortical p-Tau (preferentially in superficial layers)	–	x	x
S2: Hippocampal p-Tau	–	–	–
S3: p-Tau present in subcortical nuclei			
Mamillary bodies	–	x	x
Amygdala	–	–	–
Thalamus	–	–	–
S4: Astroglial p-Tau in subpial and periventricular regions			
p-Tau immunoreactive astrocytes in the subpial regions	–	x	x
p-Tau immunoreactive astrocytes in the periventricular regions	–	–	–
*Non-p-Tau related histological pathologies*			
S5: TDP-43 immunoreactive neuronal cytoplasmic inclusions			
Cortex	–	x	x
Hippocampus	–	–	x
Amygdala	–	–	–
Select nonspecific neuropathological features Associated with CTE*			
A1: β-amyloid precursor protein depositions	–	–	–
A2: α-synuclein depositions	–	–	–
A3: Hemosiderin laden macrophages	x	x	x
A4: Reactive microglia	–	x	x
A5: Astrogliosis	x	x	x
A6: Neuronal loss	–	x	x
A7: Axonal loss	–	x	x
A8: Blood brain barrier disruption	X	x	not done

*These selected histopathological features have been commonly reported to accompany CTE pathology but are not used to in the consensus criteria to define CTE. P, S, and A refer to pathognomonic, supportive, and associated pathology. “x “ and “–” denote histopathological feature present and absent in our model, respectively

### Neurologic evaluation

Return of the righting reflex was measured as the time (s) from TBI/sham injury to righting from a supine to prone position after discontinuation of anesthesia. The neurological severity score (NSS) was assessed on a scale from 0 (no deficit) to 10 (maximal deficit) prior to TBI as well as serially until sacrifice as previously described in detail [31].

### Statistical analysis

Unless otherwise stated, continuous variables are reported as mean ± standard error of the mean. Normality of data was examined using the Shapiro–Wilk test. One-way analysis of variance (ANOVA) on Ranks with post-hoc Dunn’s test was used to assess for between-group differences in histopathology (p-Tau, TDP-43, pTDP-43, NeuN, Iba-1, GFAP, MBP, SMI-312). Between-group comparisons of continuous variables over time (body weight, return of the righting reflex, NSS) were conducted using longitudinal mixed models. Time was treated as a categorical variable. The models included group (Sham versus rTBI) and time as fixed covariates, as well as the group × time interactions. Two-sided significance tests were used throughout and unless stated otherwise a two-sided *P* < 0.05 was considered statistically significant. All statistical analyses were performed using SigmaPlot 12.5 (Systat Software, Inc., Germany) or IBM® SPSS® Statistics Version 26 (IBM®-Armonk, NY).

### Systematic review

We conducted a systematic review of the literature by searching PubMed and Scopus using search criteria that were established to be specific for mouse models of closed head injury (including blast injuries) and that contained reference to tau protein assessment in wild type mouse strains. In addition to characterizing model characteristics of included studies, we collected histological outcomes that have been associated with CTE, including assessment of axonal and neuronal injury, astrogliosis, microglial activation, TDP-43 pathology, as well as presence of amyloid pathology, α-synuclein, cerebral microbleeds, and evidence of BBB disruption. Overall, we identified 1940 articles in PubMed and 2057 articles in Scopus. After removal of duplicates (n = 241), 3756 papers were included for screening. Details of the review methodology including the search strategy, inclusion and exclusion criteria, and retrieval of information from the full-text articles is summarized in the Additional file 1: Supplementary information.

## Results

### Mouse rTBI model

Similar to sham operated mice, brains of rTBI mice appeared macroscopically intact and without evidence for macroscopic cerebral hemorrhages at all time points post injury. Microscopically, the cerebral cortex appeared normal at 1 week after rTBI. However, at 4 and 24 weeks after rTBI we observed focal tissue disruption within the superficial cerebral cortex approximately corresponding to cortical layer I (Fig. 1c-e).

Consistent with human pathology [28], Prussian blue staining showed the presence of hemosiderin laden macrophages indicative of microvascular injury in the superficial layers of the ipsilateral cortex, at the depth of the superior longitudinal fissure, and grey-white matter junction between cortex and corpus callosum (Additional file 1: Figure S1). There was evidence of subtle BBB disruption as assessed by IgG staining at 1 week and 4 weeks post rTBI (Additional file 1: Figure S2).

### p-Tau spreads from the corpus callosum to superficial cortical layers between 1 to 24 weeks after rTBI

The primary goal of this study was to determine the spatial and temporal evolution of p-Tau pathology over 24 weeks after murine closed head-injury. We used both AT8 and AT180 to detect p-Tau. AT180 staining yielded similar results as AT8 staining (Additional file 1: Figure S3) and we exclusively refer to AT8 staining to describe p-Tau pathology below. Importantly, we found no AT8 positive cells in sham operated mice that survived for 4 weeks and 12 months after surgery, respectively (Additional file 1: Figure S4).

**Fig. 2:**
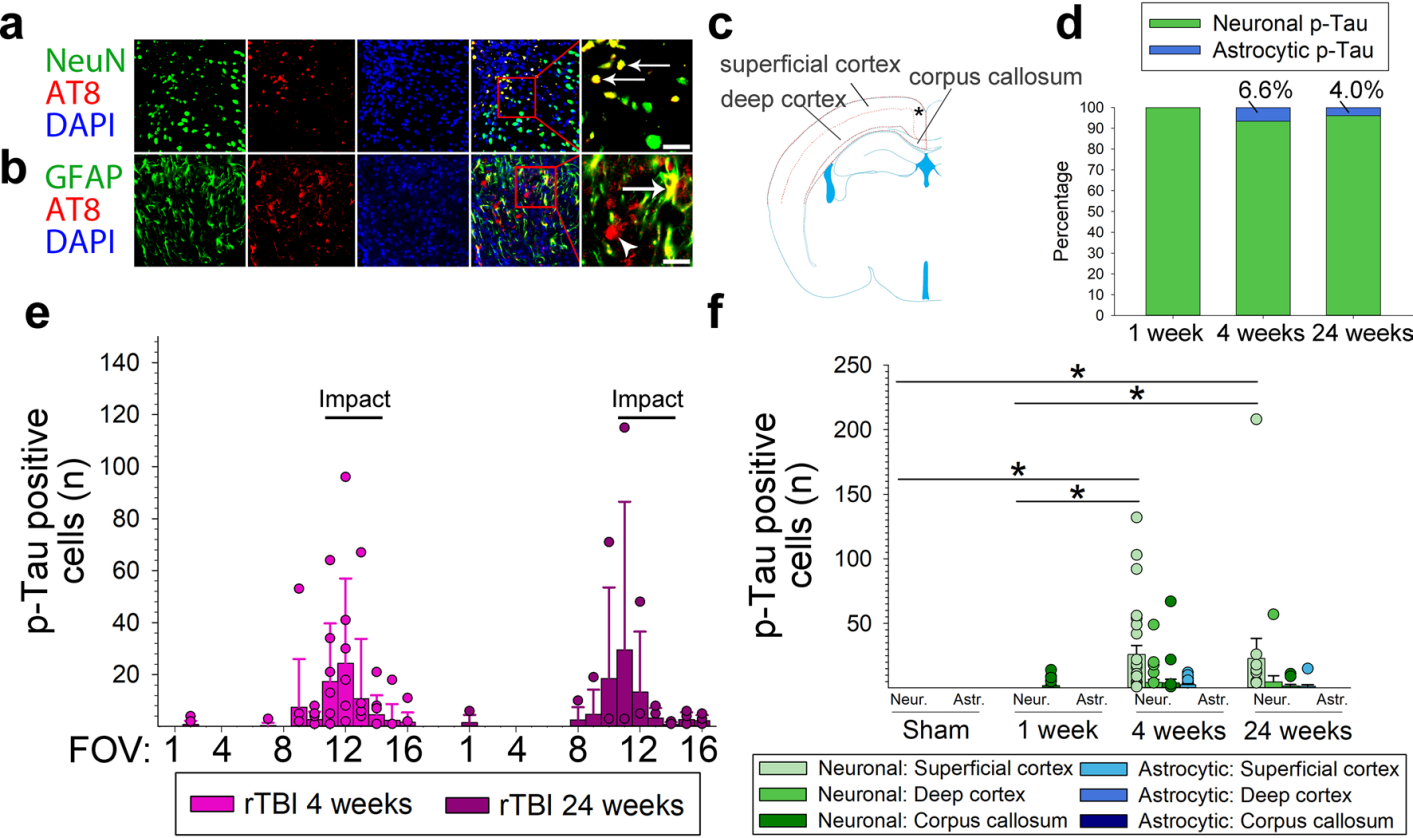
Temporal and spatial distribution of p-Tau pathology in neurons and astroglia. **(a,b)** Double staining indicates colocalization (white arrows) of hyperphosphorylated tau (p-Tau; AT8) with (**a**) neurons (NeuN) and (**b**) astrocytes (GFAP) in the cerebral cortex at 4 weeks after repetitive traumatic brain injury (rTBI). White scale bars correspond to 50 µm in low power and 17 µm in high power magnified panels. DAPI (blue) channel omitted from high power magnifications. (**c**) Dotted lines delineate cortical layers I-III (superficial cortex) from cortical layers IV-VI (deep cortex) and corpus callosum used to assess the presence of p-Tau stained cells in the ipsilateral hemisphere. Asterisk denotes the approximate location of the images taken for **a-b**. **(d)** Proportion of AT8 positive neurons and astrocytes at the investigated time points after rTBI. **(e**) Distribution of AT8 stained cells in the cerebral cortex (superficial + deep combined) relative to the impact center (black bars). There was no difference in the number and distribution of p-Tau positive cells at 4 weeks and 24 weeks after rTBI (*P* > 0.05). Because sham and 1-week rTBI animals had no cortical AT8-positive cells they were omitted from this analysis. Each bar corresponds to one cortical field of view (FOV) arranged from left, contralateral (FOV 1) to right, ipsilateral (FOV 16), whereby corresponding FOVs in the three investigated sections s1–s3 were summed. Data are mean ± SD. **(f)** Number of p-Tau positive neurons (green shades) and astrocytes (blue shades) in the traumatized hemisphere stratified by location in the superficial cortex, deep cortex, and corpus callosum over time (total number of cells counted in the three investigated sections s1–s3). *P < 0.05. Data are mean ± SEM. n = 8 mice for sham, 1 week, and 4 weeks, n = 4 for 24 weeks (there were no p-Tau positive cells in sham operated mice). All analyses were done using one-way ANOVA on Ranks with post-hoc Dunn's

We found that 1 week after rTBI, 4 mice (n = 50%) showed faint AT8 staining restricted to the corpus callosum without any p-Tau accumulation in the overlying cerebral cortex (Figs. 1c, f, 2f). P-Tau pathology did not substantially progress in the corpus callosum between 1 and 24 weeks and AT8 positive cells were present in only a subset of mice (n = 3 [38%] at 4 weeks, n = 2 [50%] at 24 weeks), typically within the section adjacent to the impact center (Figs. 1b, f–h, 2f). Conversely, by 4 and 24 weeks we observed significant accumulation of AT8-stained cells in all investigated mice, particularly in subpial locations and the superficial cortex (approximately cortical layers I-III) in the traumatized hemisphere (Figs. 1d–e, 2e–f). Figure 1b depicts the approximate distribution of AT8-positive cells within the traumatized hemisphere at 4 weeks after rTBI. Interestingly, staining was most abundant in the cerebral cortex adjacent to the impact site (Bregma − 1.67 mm) rather than directly below the impact center (Bregma −3.07).

### p-Tau accumulation after murine rTBI occurs in cerebral locations reported for human TBI-associated tauopathy

At 4 weeks post rTBI we observed perivascular p-Tau accumulation (Fig. 1i, m), a pathognomonic histological feature of CTE. Additional, though less common, sites of p-Tau accumulation included the mamillary body (Fig. 1l) and periventricular tissues (Fig. 1k, l), which are considered histological features supporting a CTE diagnosis. Notably, in all examined rTBI mice p-Tau accumulation was present in the subpial cortex adjoining the superficial longitudinal fissure (Fig. 1b, m). We did not find any AT8 stained cells in the hippocampus, thalamus, and amygdala (Table 1).

### p-Tau accumulates in cortical neurons and astrocytes

We found distinct co-localization of AT8 with both NeuN and GFAP in the traumatized hemisphere consistent with neuronal and astrocytic tau accumulation (Fig. 2a, b). Interestingly, colocalization of AT8 and GFAP was only observed at 4 and 24 weeks after rTBI and restricted to the superficial cerebral cortex without overt co-staining in deeper cortical layers or the corpus callosum (Fig. 2d, f). We found that perivascular p-Tau was present in both neurons and astrocytes (Additional file 1: Figure S5). The majority of AT8 positive cells co-stained with NeuN, and less than 10% of cells were positive for both AT8 and GFAP (Fig. 2d, f).

### rTBI causes persistent nuclear loss and cytoplasmatic localization of TDP-43 and pTDP-43

**Fig. 3:**
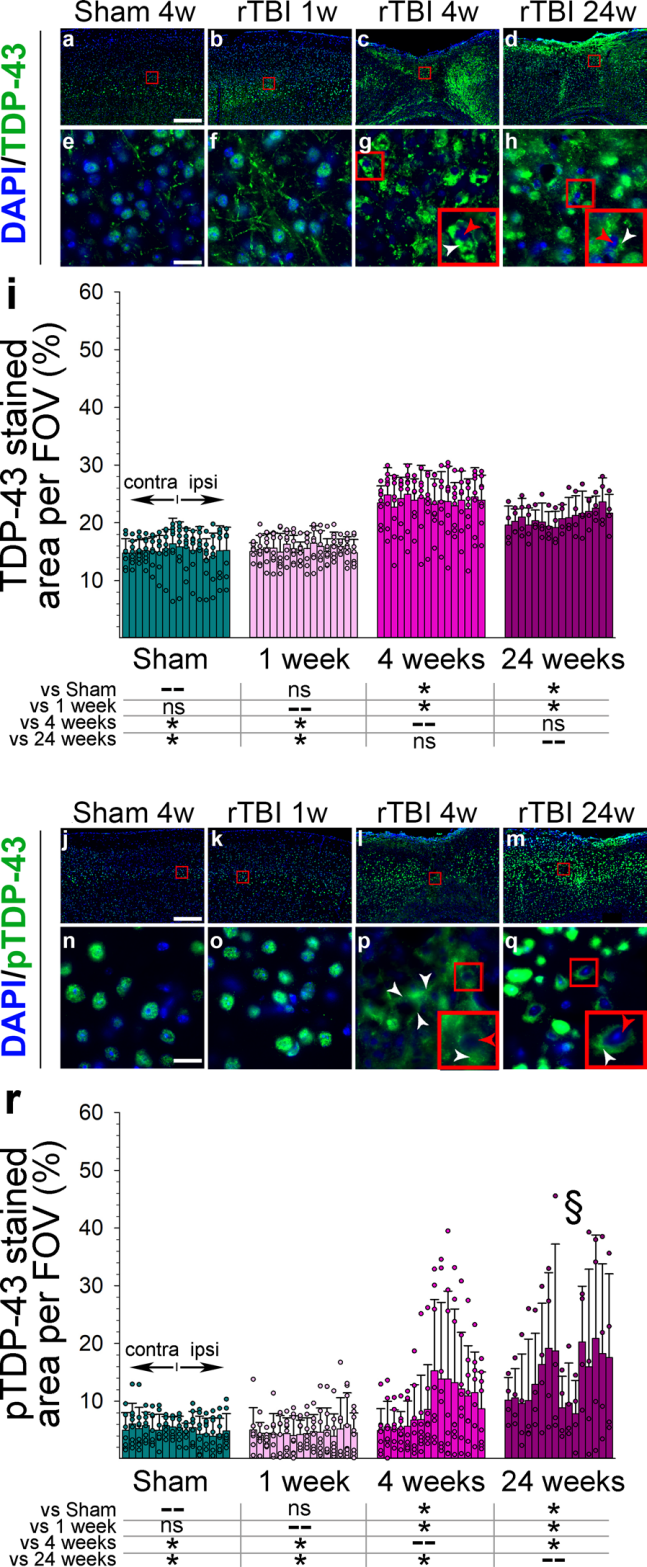
Patterns and evolution of TDP43 (**a–h**) and pTDP43 (**j–q**) pathology in the cerebral cortex after repetitive closed head traumatic brain injury (rTBI) in the mouse. (**f**) Increased linear pattern of TDP-43 reactivity suggesting neuritic distribution at 1 week after rTBI. Nuclear loss (red arrowheads) and cytoplasmatic localization (white arrowheads) of TDP-43 (**g-h**) and pTDP-43 (**p-q**) at 4 and 24 weeks. Aggregation of cytoplasmatic localized TDP-43 (**g-h**) and pTDP-43 (**q**). (**r**) Apparent reduction in signal intensity of pTDP-43 in the cortex next to the impact center (§). Scale bars correspond to 240 µm for low power and 20 µm for high power magnified images. In each group, bars correspond to one cortical field of view (FOV) arranged from left, contralateral (contra), to right, ipsilateral (ipsi). Data are shown as mean (+ SD). n = 8 for sham, 1 week, and 4 week post rTBI and n = 4 for 24 week post rTBI. Analyses were done at Bregma − 1.67 mm (corresponding to s2 in Fig. 1) *P < 0.05. All analyses were done using one-way ANOVA on Ranks with post-hoc Dunn's

Compared to sham and 1 week post rTBI mice, we noted an overall increase in TDP-43 and pTDP-43 in the ipsilateral cerebral cortex, particularly adjacent to the impact center, at 4 weeks and 24 weeks after rTBI (Fig. 3). On a cellular level, there was nuclear loss and cytoplasmatic localization of TDP-43 and pTDP-43 at the same time points (Fig. 3). However, while conspicuous aggregation of cytoplasmatic TDP-43 was present at 4 weeks (Fig. 3g), this was only observed at 24 weeks for pTDP-43 (Fig. 3q).

In the contralateral (non-traumatized) hemisphere staining intensity for TDP-43 and pTDP-43 in the corpus callosum, subcortical nuclei, and hippocampus was overall similar to sham operated animals at all time points after rTBI (not shown). Nonetheless, by 24 weeks after rTBI, nuclear loss and cytoplasmic localization of TDP-43 (Additional file 1: Figure S6) and pTDP-43 (not shown) was present in the contralateral cerebral cortex and bilateral CA1 of the hippocampus.

### rTBI causes astroglial and microglial activation and progressive neurodegeneration

In addition to assessing pertinent p-Tau and TDP-43 related histological features of human CTE (Table 1), we sought to determine pathology that occurs in human TBI-associated neurodegenerative disease, including the presence of reactive migroglia and astroglia, neuronal and axonal loss, and accumulation of βAPP and α-synuclein.

**Fig. 4:**
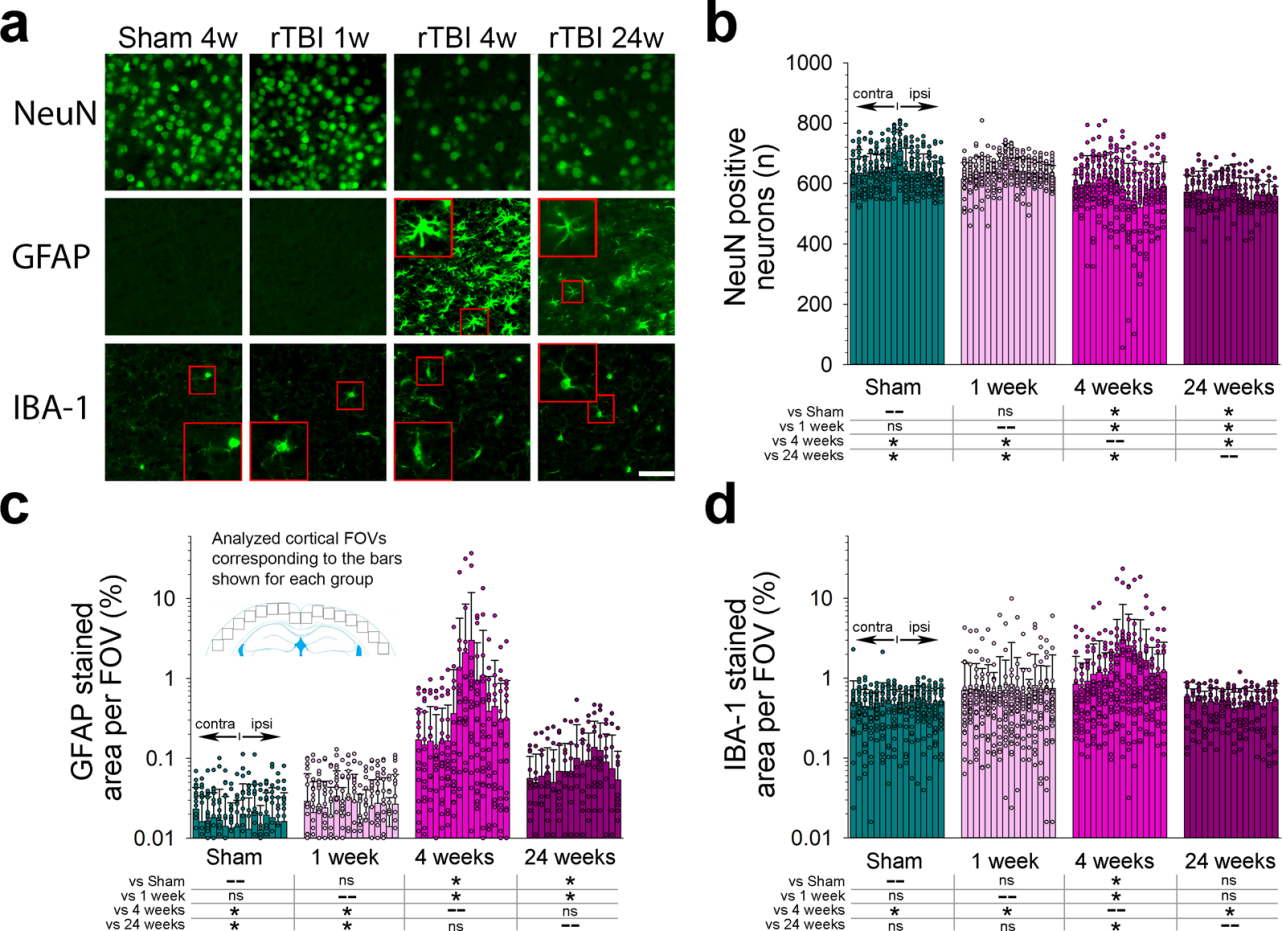
Progression of cortical neuronal loss and glial activation after repetitive traumatic brain injury (rTBI). **(a)** Cortical neuronal loss after rTBI as shown by NeuN staining. (**b**) Astroglial activation at 4 weeks as indicated by (**a**) the presence of numerous hypertrophied GFAP stained astrocytes (inset) and (**c**) a significant increase in GFAP staining signal. Decrease in astroglial activation at 24 weeks after rTBI as shown by reduced cell hypertrophy (inset in (**a**)) and attenuated GFAP staining (**c**). Microglial activation was observed at 4 weeks as indicated by the presence of numerous bipolar/rod shaped Iba-1 stained microglia (inset in (**a**)) and significantly increased in Iba-1 stained area (**d**). Normalization of microglial activation at 24 weeks after rTBI as noted by increased ramification of Iba-1 stained cells (inset in (**a**)) and similar Iba-1 staining intensity as compared to sham operated mice (**d**). Each bar corresponds to one cortical field of view (FOV) arranged from left, contralateral (contra), to right, ipsilateral (ipsi). Data are shown as mean (+ SD). n = 8 (sham, 1 week and 4 week post rTBI, each) and n = 4 (24 week post rTBI) mice. Analyses are based on three coronal sections (corresponding to s1–s3 in Fig. 1). *P < 0.05. All analyses were done using one-way ANOVA on Ranks with post-hoc Dunn's

Sham and 1-week rTBI animals had few GFAP positive cells in the cerebral cortex and corpus callosum (< 1% staining signal coverage) without difference between hemispheres (Fig. 4c). We observed substantial astroglial activation at 4 weeks after rTBI as indicated by the presence of hypertrophied GFAP stained astrocytes and overall significantly increased GFAP staining in the cerebral cortex (Fig. 4a, c). Although GFAP staining remained significantly elevated by 24 weeks as compared to sham operated animals, overall staining intensity appeared attenuated, and cells appeared less hypertrophied when compared to 4 weeks (Fig. 4a, c).

There was significant microglial activation in the cerebral cortex at 4 weeks after rTBI as indicated by the presence of bipolar/rod shaped Iba-1-stained microglia and overall increased Iba-1 staining (Fig. 4a, d). Activation subsided by 24 weeks as indicated by a more ramified appearance of Iba-1-stained cells and return of Iba-1 staining intensity to sham levels (Fig. 4a, d).

At 1 week after rTBI, neuronal density in the ipsilateral cortex was similar to sham operated mice (Fig. 4a, b). However, by 4 weeks after rTBI, we observed a significant loss of NeuN profiles, which worsened by 24 weeks indicative of progressive neurodegeneration (Fig. 4a, b). Loss of NeuN positive cells was most pronounced within the ipsilateral cerebral cortex adjacent to the impact site (rather than beneath the impact center) corresponding to the area of maximal p-Tau accumulation (compare with Fig. 1a).

**Fig. 5:**
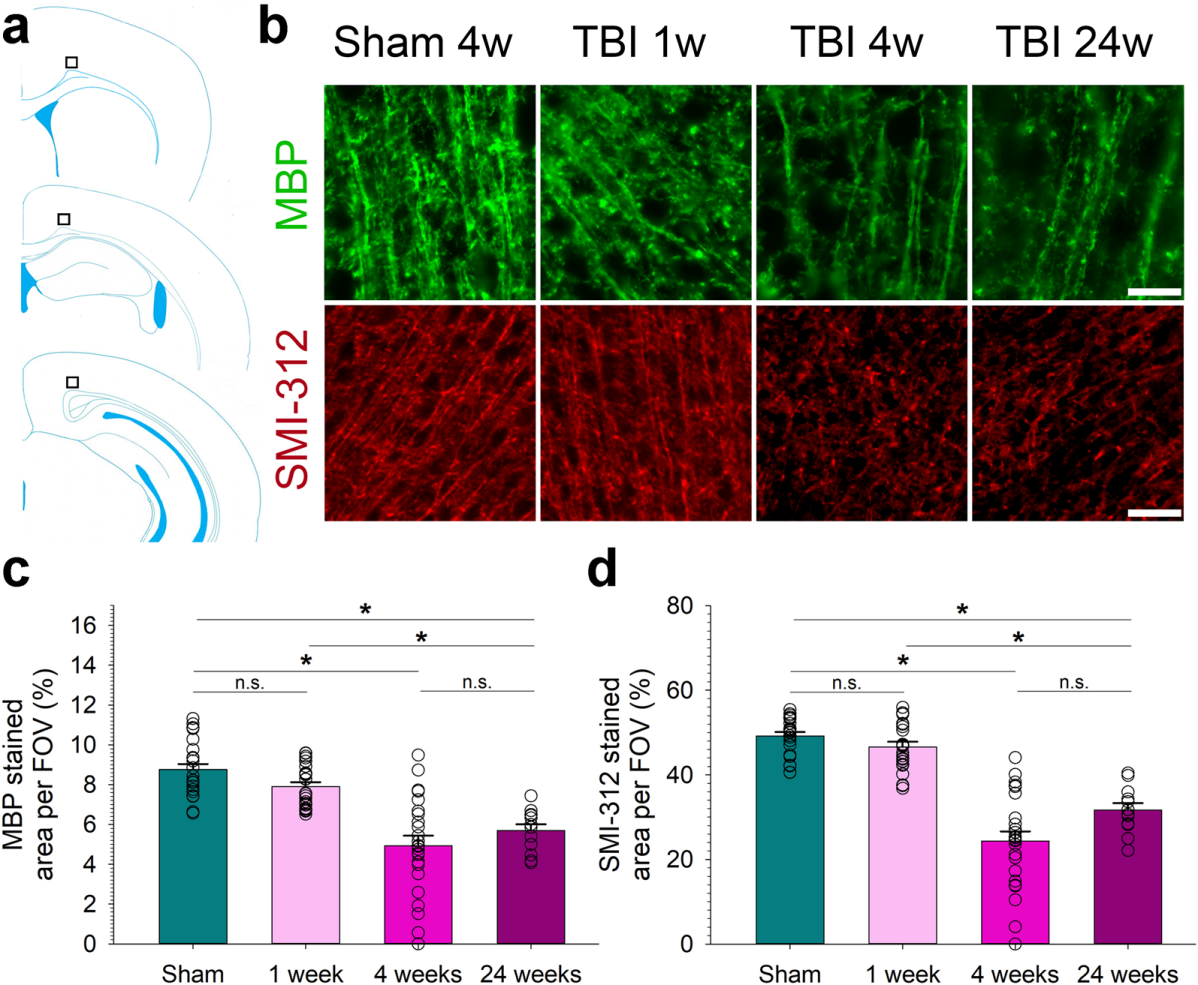
Axonal loss in cortex following repetitive traumatic brain injury (rTBI). (**a**) Cartoon depicting the 3 sections s1–s3 used for analysis. Black boxes indicate the location of the three fields of view (FOVs) used to quantify the immunofluorescent imaging signal for the myelin marker myelin basic protein (MBP) and the pan-axonal neurofilament marker SMI-312. (**b**) Representative photomicrographs showing progressive loss of the MBP and SMI-312 staining signal from 1 to 24 weeks after rTBI. Quantified staining signal for (**c**) MBP and (**d**) SMI-312. Data are mean ± SEM. n = 8 mice for sham, 1 week, and 4 weeks, n = 4 for 24 weeks. *P < 0.05. n.s. indicates not significant. All analyses were done using one-way ANOVA on Ranks with post-hoc Dunn's. Scale bar is 50 µm

At 1 week after rTBI, MBP and SMI-312 staining in the traumatized cerebral cortex appeared morphologically similar without significant difference in the quantified signal intensity as compared to sham operated mice (Fig. 5b). Concurrent with neuronal injury, we observed a significant loss of MBP and SMI-312 stained profiles indicating axonal degeneration by 4 weeks and 24 weeks after rTBI (Fig. 5c, d).

Lastly, we found no βAPP and α-synuclein depositions in any of the operated mice (not shown).

### rTBI causes long-term functional deficits

**Fig. 6:**
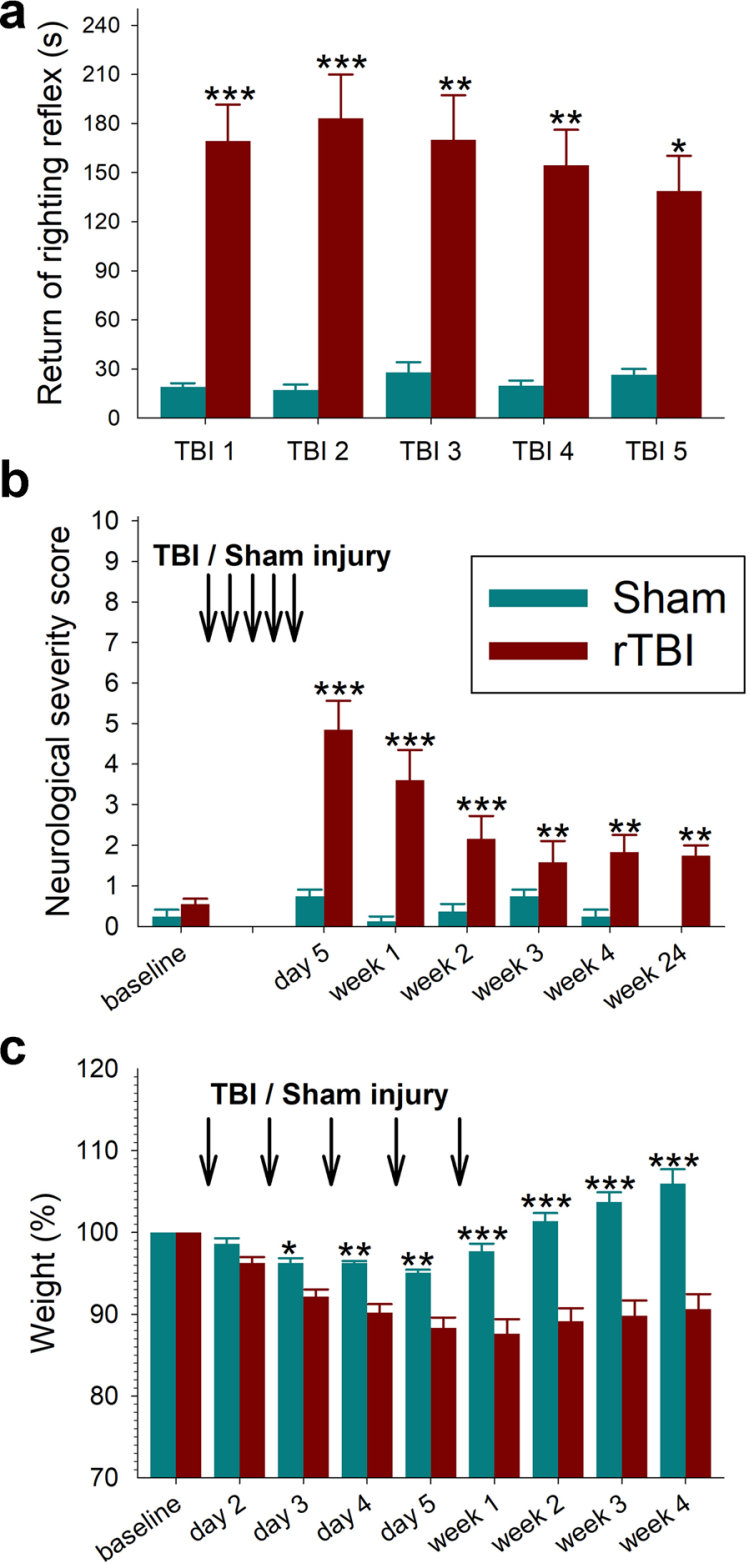
Persistent neurological deficits in mice subjected to repetitive traumatic brain injury (rTBI). **(a)** Significantly prolonged time to the return of the righting reflex (RR) after discontinuation of anesthesia in mice subjected to repetitive TBI (rTBI) versus sham operated animals. There was as a significant group (*P* < 0.001) effect but no significant time (P = 0.958) effect or a group x time (P = 0.574) interaction (*P < 0.05, **P < 0.01, ***P < 0.001 versus sham). **(b)**. There were significant group (P = 0.003) and time (*P* < 0.001) effects as well as presence of a significant group x time interaction (*P* < 0.001) for the composite neurological severity score (**P < 0.01, ***P < 0.001 versus baseline). **(c)** There were significant group and time effects as well as presence of a significant group x time interaction (*P* < 0.001, each) for the change in body weight during the first 4 weeks after surgery (*P < 0.05, **P < 0.01, ***P < 0.001 versus sham). Data are mean ± SEM. All statistical comparisons were made using mixed effects models (n = 8 for sham; n = 20 for baseline to 1 week after rTBI, n = 12 for 2 to 4 weeks after rTBI, and n = 4 for 24 weeks after rTBI). For clarity in the figure, *post-hoc* pairwise comparisons are only shown for significant differences between groups (**a, c**) and versus baseline (**b**)

Sham operated mice regained their righting reflex within a median of 24 s (interquartile range 14–32 s) after discontinuation of anesthesia. In contrast, righting reflex was significantly suppressed in rTBI mice and only returned after a median of 135 s (interquartile range 79–229 s) (Fig. 6a).

We used the NSS, which is a composite of ratings measuring a combination of overall inquisitiveness, postural stability, and motor function, to examine the temporal evolution of functional deficits in rTBI animals versus controls. Whereas sham operated animals had no change in the NSS over the observation period, rTBI mice had significant neurological deficits at 2 h after the last impact (day 5) when compared to baseline and controls. Although deficits partially improved over time, persistent residual neurological deficits were present by 24 weeks (Fig. 6b).

Although both sham and rTBI mice lost weight after surgery, sham injured animals regained their baseline weight by 1 week whereas rTBI mice had a persistent weight loss up to 4 weeks after rTBI (Fig. 6c).

### Systematic literature review

#### p-Tau pathology is infrequently reported in murine closed head models

From searches on both PubMed and Scopus, a total of 3997 articles were initially included. After removal of 241 duplicates 3756 articles were screened for inclusion and exclusion criteria (Additional file 1: Figure S7a). From this screening, we excluded 2394 articles because they did not include the predetermined keywords and 411 studies that did not use mice, leaving 951 articles included in the full text search. After full text review, we excluded studies that were not published in a peer-reviewed journal (n = 5); review articles (n = 235); conference proceeding without primary data (n = 30); studies using transgenic animals without wild-type mice (n = 106); lacking assessment of cerebral tau (n = 507); and penetrating head injury (n = 12). Lastly, two studies identified from references were added, resulting in a total of 58 studies (1.5% of screened publications) that investigated the presence of pathological tau in the brains of mice subjected to closed-head injury.

### Characteristics of included studies reporting tau pathology assessment

Among the 58 included studies, 114 different injury paradigms were used (42 single TBI and 72 repetitive TBI models). Most models utilized male mice (112/114 [98%]), the C57BL/6 strain (111/114 [97%]), and anesthesia (111/114 [97%]). The median mouse age at the time of injury was 12 weeks (range 5 to 48 weeks). The most commonly used models were weight drop (n = 51), piston driven (n = 42), blast injury (n = 12), and rotational impact acceleration (n = 9) models.

Additional file 1: Figure S7b shows the primary methodologies used for p-Tau detection and the frequency of p-Tau detection. Of the included studies, 40 (68%) reported p-Tau pathology and 25 (44%) described p-Tau pathology by immunostaining (Additional file 1: Figure S7c-d). The most commonly used antibody to detect p-Tau was AT8 (8/25 [32%] of studies). Of the 25 studies that observed tau pathology, 22 found p-Tau in the cerebral cortex, hippocampus, or subcortical structures and 3 studies reported the presence of p-Tau in the optic tract (n = 2) and cerebellum (n = 1). Table 2 summarizes key characteristics of the 25 TBI models (15 repetitive TBI and 10 single TBI) that were associated with cortical or hippocampal p-Tau, as assessed by immunohistochemistry.

**Table 2:** Mouse traumatic brain injury (TBI) models reporting p-Tau histopathology in the brain

Reference	Model	Impacts	ITI	EDPI	p-Tau Ab	CTE-like pathology*		
*Repetitive TBI*						Pathognomonic	Supportive	Associated
This study	WD	5	24	7	AT8	P1, P2	S1,S3,S4, S5	A3 to A8
Petraglia et al. [54]**	PD	6	24	30	AT8	P1, P2	S1,S2,S3,S4	A4, A5
Shin et al. [64]	WD	20	24	42	P(T205)	P1	S1, S2, S4	A5
Zhang et al. [78]	PD	3	24	8	AT8	–	S1, S2, S5	A1, A4, A5, A6, A7
Luo et al. [44]	PD	3	24	180	AT8	–	S1, S2, S3	A5
Briggs et al. [13]	WD	30	24	93	AT8	–	S1, S5	A1, A4, A5, A7
Albayram et al. [1]**	RIA	7	24	14	Cisptau	–	S1, S5	A1, A4,A5, A7
Selvaraj et al. [62]	PD	3	24	8	P(S404)	–	S1, S2	A1, A4, A5, A6
Kondo et al. [40]**	RIA	7	24	10	Cisptau	–	S1, S2	A6, A7
Albayram et al. [2]	RIA	5	24	240	AT8	–	S1, S2	A4
Yang et al. [76]	PD	4	72	10	CP13	–	S1, S2	A5
Sacramento et al. [58]	PD	10	2	21	AT8	–	S1,S2	–
Tagge et al. [69]	RIA	2	0.25	1	Cisptau	–	S1	A4, A5, A7, A8
Rehman et al. [56]	WD	3	24	7	P(S413)	–	S1	A1, A6, A8
Seo et al. [63]	WD	3	72	7	PHF	–	S2	–
Niziolek et al. [51]	WD	4	48	30	P(S262)	–	S2	–
***Single TBI***								
Liu et al. [42]	RIA	1	n/a	30	T231	P1	S1, S2	–
Petraglia et al. [54]**	PD	1	n/a	30	AT8	–	S1, S2, S3	A5
Goldstein et al. [26]	PD	1	n/a	14	CP13	–	S1,S2	A4, A5, A6, A7
Kondo et al. [40]**	RIA	1	n/a	1	Cisptau	–	S1, S2	A6, A7
Huber et al. [35]	Blast	1	n/a	30	CP13	–	S1,S2	A6
Sabbagh et al. [57]	Blast	1	n/a	56	T231	–	S1, S2	–
Iliff et al. [37]	PD	1	n/a	28	AT8	–	S1	A4, A5, A7
Albayram et al. [1]**	RIA	1	n/a	14	Cisptau	–	S1	A1, A7
Logsdon et al. [43]	Blast	1	n/a	3	T22	–	S1	A5,A8
Niziolek et al. [50]	WD	1	n/a	30	P(S262)	–	S2	A4

*See Table 1 for definitions. **Used both single and repetitive TBI. EDPI indicates the earliest day post injury at which p-Tau was found after TBI. If more than one p-Tau Ab was used only the antibody used to depict the main results is indicated. ITI, inter-injury-interval (hours), n/a, not applicable; PD, piston driven; RIA, rotational impact acceleration; WD, weight drop. Four studies reporting p-Tau exclusively in the optic tract and cerebellum are excluded from this table. P, S, and A refer to pathognomonic, supportive, and associated pathology (see Table 1 for details). Dashes indicate histopathological feature not reported or not found. Studies not showing p-Tau in the cerebral cortex or hippocampus are omitted from this table

### Presence of CTE-like pathology in mouse models reporting tau pathology

With respect to the presence of CTE-like pathology, only 3 studies (5.2% of included and 0.1% of all screened papers) reported perivascular p-Tau, a defining feature of human CTE. One of these studies specifically mentioned the presence of both neuronal and astroglial p-Tau (Additional file 1: Figure S7c). One additional study reported concurrent p-Tau pathology in both astroglia and neurons (located in the cortex and hippocampus) but did not report on potential perivascular location.

In terms of non-p-Tau related supportive histopathological CTE-like features, 3 studies reported the presence of cytoplasmatic localization of TDP-43 pathology (none specifically commented on pTDP-43) (Additional file 1: Figure S7c).

Forty-six (79%) of the included studies reported non-specific CTE-associated pathologies (Additional file 1: Figure S7d). Most commonly, these included neuronal loss (23%), axonal loss (43%), micro- and astrogliosis (50%, each), and beta amyloid deposition (29%) (Additional file 1: Figure S7c). Interestingly, no study reported cerebral microbleeds.

## Discussion

Pathological hyperphosphorylation and aggregation of Tau protein is observed in a wide range of neurodegenerative disorders and is the key defining feature of a heterogeneous class of diseases called tauopathies. TBI has been identified as a strong risk factor for many of these tauopathies, highlighting the potential lifelong consequences of TBI exposure. Recently, CTE has been described as the prototypical TBI-associated tauopathy. Its diagnosis presently rests with the unique distribution of tau pathologies on a macroscopic and cellular level, yet little is known to what extent these pathologies are replicated by mouse closed-head TBI. To close this knowledge gap we described the pertinent histopathological features of human TBI-associated tauopathy in our rTBI model as well as by conducting a systematic review of the literature.

Our concussive mouse rTBI model showed p-Tau accumulation within the corpus callosum of the traumatized hemisphere as early as 1 week after injury with subsequent involvement of the cerebral cortex, mimicking the pathological tau progression described in human disease. Importantly, we found p-Tau in multiple cerebral locations that are frequently involved in human disease and are included in the CTE consensus criteria. These included, superficial cortical layers, the hippocampus, periventricular tissues, mamillary bodies, and perivascular locations [8, 46]. Nevertheless, we defined vessels based on morphology only and future studies should include staining for vascular markers to determine the specific association of p-Tau with the vascular compartment.

In this regard, it should be noted that human CTE criteria include the location of perivascular p-Tau at the depth of cerebral sulci [8, 46]. Because mice are lisencephalic, this criterion cannot be fulfilled in a murine model. However, we consistently observed p-Tau within the subpial cerebral cortex lining the superficial longitudinal fissure, as well as in the adjacent corpus callosum. This is consistent with computational models of the biomechanical forces predicting that cerebral areas with a change in morphology (such as at the site of sulci and the superior longitudinal fissure) represent locations of high stress and strain and thus greatest susceptibility to axonal and vascular injury [12, 16, 23].

Given the perivascular location of many neurodegenerative features it has been suggested that TBI triggers the neurodegenerative cascade by damaging the neurovascular unit. Indeed, consistent with human data [28, 39], we observed microvascular injury in our model, particularly in locations with p-Tau accumulation. Moreover, using IgG staining, we found evidence for BBB-disruption adding to the notion that BBB hyperpermeability is an important aspect of murine TBI-associated tauopathy. Nevertheless, IgG staining was faint and restricted to the optic tract. Injury to the visual pathway including BBB-disruption and p-Tau accumulation in the optic tract has been shown after murine closed head injury [3, 13, 14, 19]. Yet, we did not observe previously reported perivascular IgG staining in the cerebral cortex [69]. This is inconsistent with the observed microvascular injury as assessed by Prussian blue staining in our study. In this respect it is noteworthy that we used the cyclooxygenase-2 (COX-2) inhibitor carprofen as a post-operative analgesic. Carprofen has been shown to attenuate microglial activation, inflammation, and brain edema formation at therapeutic doses in mice, which may have attenuated BBB-disruption in our model [72]. Thus, while IgG-staining provided proof-of-principle that BBB-integrity was impaired in our model, further studies using different BBB-integrity markers as well as avoidance of anti-inflammatory drugs is required to better characterize the extent of BBB permeability and its relationship to p-Tau and other observed pathologies.

In addition to pathological p-Tau accumulation, several other proteinopathies have been described following human TBI. In particular, widespread TDP-43 inclusions in the neocortex of patients with CTE have been reported and the presence of TDP-43 pathology has been used to support the diagnosis of CTE [47, 49]. Nevertheless, TDP-43-related pathology is not specific to CTE. It has been described in a range of conditions, and colocalization of tau and TDP-43 is often limited. For this reason, it has yet to be determined exactly how TDP-43 aggregates coincide and interact with pathological p-Tau accumulation. Overall, few mouse studies have sought to evaluate TDP-43 pathology after closed-head TBI [1, 13, 73, 78]. Here, we found widespread TDP-43 expression following rTBI with a persistent presence of cytoplasmatic mislocalization by 6 months post rTBI. This is consistent with prior studies reporting persistent TDP-43 pathology in murine TBI associated with tauopathy [1, 13, 78]. We found long-term accumulation of pTDP-43 expression with associated nuclear loss and cytoplasmatic aggregation up to 24 weeks after rTBI. This is an important extension of previous studies that were limited to shorter (1 week) observation periods [55, 70, 73] and in light of reported transient alterations [55, 73]. Our observation that pathological TDP-43, but not tau, accumulation was present in the hippocampus adds to the notion that while these proteinopathies share a common pathophysiology, there may exist cell-specific susceptibilities [1, 68].

In contrast to pathological tau and TDP-43 accumulation after TBI, persistent α-synuclein and β-amyloid proteinopathy represent less consistent histopathological features after TBI [7, 8, 22, 38, 67]. Although several prior animal studies reported increased β-amyloid and α-synuclein after TBI, we did not find this present in our rTBI model [1, 13, 56, 62, 75, 78].

Mixed neuronal and astroglial tau pathology is considered a hallmark of CTE; as such, it is important to evaluate the precise cell types involved with TBI-associated tauopathy. For example, astrocyte activation is common after TBI, accompanies virtually all neurodegenerative tauopathies, and astrocytes may serve as a source for tau and thus could conceivably contribute to pathological tau accumulation [34, 41, 65, 69]. Yet, whether astroglial tau expression serves as a driver for injury-associated tauopathy remains uncertain [41]. Many groups hypothesize that astrocytes may promote neurodegeneration because astroglial tau pathology has been observed in the absence of neuronal tau inclusions, possibly through pro-inflammatory mechanisms [41]. We observed significant neuronal loss by 4 weeks after rTBI, which coincided with significant astroglial and microglial activation. However, we noted that neuronal p-Tau expression occurred prior to significant microglial and astroglial activation. Moreover, neuronal p-Tau expression was present earlier and progressed more widespread than astroglial p-Tau expression. Lastly, p-Tau-stained astrocytes were located in the superficial cortex, which had the greatest burden of neuronal p-Tau, but there were no p-Tau expressing astrocytes in deeper cortical layers and the corpus callosum. Together, these observations are consistent with the hypothesis that astroglial p-Tau expression after closed-head TBI is a secondary event rather than primary driver of tau pathology, possibly related to internalization of p-Tau into astrocytes from neighboring neurons and synapses [41].

To put our results in context with ongoing work in the field, we conducted a systematic review of the literature. Our analysis showed a striking paucity of studies that sought to determine tau pathology in experimental closed-head mouse TBI. Fewer than 2% of screened studies (58/3,756) sought to assess pathological tau accumulation. Nevertheless, of these studies, approximately 40% found evidence of p-Tau pathology by immunohistochemistry. Despite the overall scarcity of investigations, these and our observations provide cumulative evidence that murine closed head TBI replicates the critical histopathological aspects of human TBI-associated tau pathology with specific features of CTE. Reflecting human disease, pathology was seen across a wide variety of TBI-paradigms, highlighting that tau pathology after murine closed head TBI is reproducible and robust. This indicates that model differences could be leveraged to study the association of pathophysiological mechanisms with varying mechanics of injury [9] as well as the ability to control for possible confounders. For example, similar to other studies reporting p-Tau accumulation after murine closed-head rTBI [56, 62, 75, 78] we exposed the skull for precise impact delivery to the same coordinates across animals. Yet, this is inconsistent with the clinical situation, and our systematic review showed that this approach is not critical for inducing pertinent CTE-like neuropathological features.

Finally, though accumulation of p-Tau is a critical early event in the cascade leading to tauopathy, formation of small, soluble oligomeric tau species as well as the aggregation into larger insoluble filaments known as neurofibrillary tangles (NFTs), represent the hallmark of tauopathies including CTE [5, 30, 33]. There is strong evidence that pathogenic tau, sometimes referred to as a tau prion or prion-like tau, self-templates to progressively spread disease in tauopathy patients. In a sub-set of rTBI patients, the repeated injury gives rise to the formation of tau prions, which include a toxic species responsible for neuronal death. However, further studies are needed to determine if murine closed-head models are able to replicate the formation of tau prions in mice [24, 25, 33].

In addition to ongoing efforts to better understand the effects of TBI in wild-type mice, several groups have used transgenic mouse models expressing human tau to investigate the link between traumatic injury, tau misfolding, and p-Tau pathology. For example, the rTg4510 mouse model, which expresses a doxycycline-repressible isoform of tau containing the P301L mutation [59] developed elevated p-Tau levels in the cortex 7 days after either a single or double closed-head injury [6]. Investigating the longer-term effects of TBI on tau aggregation in Tg mice, others have used the PS19 mouse model [77], which expresses human tau with the P301S mutation, and performed a semi-quantitative analysis of p-Tau pathology up to 7 months post-injury [71]. While these studies have helped address several questions about the link between TBI and tau spreading, there are also important caveats to consider with regard to these mouse models. A careful analysis of the transgene insertion sites in the rTg4510 model revealed that disruption of essential genes is at least partially responsible for the p-Tau pathology that results from overexpressing the human protein [21]. And extensive variability in the PS19 model, with regard to both the rate of tau spreading and p-Tau formation [74], limits the interpretations that can be drawn from studies using the mouse line.

In conclusion, our study highlights the translational value of murine closed skull TBI to replicate the pertinent aspects of human TBI-associated tauopathy with a wide range of related histopathological features.

## Supplementary Information

**Additional file 1**. Supplementary information.

## Data Availability

Data supporting the findings of this study are available from the corresponding author upon reasonable request.
