# Supplementary material for: Mouse closed head traumatic brain injury replicates the histological tau pathology pattern of human disease: characterization of a novel model and systematic review of the literature
Source: Acta Neuropathol Commun. 2021 Jun 29;9:118. doi: 10.1186/s40478-021-01220-8 (PMC8243463; doi:10.1186/s40478-021-01220-8)
Supplement: Supplementary file 1 — Additional file 1. Supplementary information. [file 40478_2021_1220_MOESM1_ESM.pdf]

Supplementary Information

Title: Mouse closed head traumatic brain injury replicates the histological tau pathology pattern of human disease. Characterization of a novel model and systematic review of the literature

Authors: Aydan Kahriman[1], James Bouley[1], Thomas W Smith[2], Daryl A. Bosco[1], Amanda L. Woerman[3], Nils Henninger[1,3]

Affiliations:

[1]: Department of Neurology, University of Massachusetts Medical School, Worcester, MA 01655, United States

[2]: Department of Pathology, University of Massachusetts Medical School, Worcester, MA 01655, United States

[3]: Department of Biology, University of Massachusetts Amherst, Amherst, MA 01003, United States

[4]: Department of Psychiatry University of Massachusetts Medical School, Worcester, MA 01655, United States

***Correspondence to:** Nils Henninger, MD, PhD, Dr med
Departments of Neurology and Psychiatry
University of Massachusetts Medical School
55 Lake Ave, North
Worcester, MA 01655
Tel: (774) 455-3760
Fax: (508) 856-2811
E-mail: nils.henninger@umassmed.edu

Inventory of Supplemental Information

a) Supplemental Methodology for the Systematic review

b) Supplemental Figures and Legends

c) ARRIVE Checklist

d)PRISMA-S Checklist

Supplemental Methodology for the Systematic review

Search strategy

We searched PubMed and Scopus on 2/21/2021 using search criteria that were established to be specific for mouse models of closed head injury (including blast injuries).

For seach in PubMed we used (((“chronic traumatic encephalopathy” OR “mild traumatic brain injury” OR “concussion” OR “repetitive brain trauma” OR “repetitive head impacts” OR “traumatic brain injury” OR “closed head trauma” OR “closed head injury”) AND “mice“) NOT (CCI[tiab] OR “controlled cortical impact”[tiab] OR “fluid percussion”[tiab])).

For search in Scopus we used ((chronic AND traumatic AND encephalopathy) OR (mild AND traumatic AND brain AND injury) OR (concussion) OR (repetitive AND brain AND trauma) OR (repetitive AND head AND impacts) OR (traumatic AND brain AND injury) OR (closed AND head AND trauma) OR (closed AND head AND injury)) AND NOT ((cci) OR (controlled AND cortical AND impact) OR (fluid AND percussion)) AND (LIMIT-TO (EXACTKEYWORD, “Animals”) OR (LIMIT-TO (EXACTKEYWORD, “Mice”) OR (LIMIT-TO (EXACTKEYWORD, “Mouse”)). We identified 1940 articles in PubMed and 2057 articles in Scopus. After removal of duplicates (n=241), 3756 papers were included for screening.

Inclusion and exclusion criteria

Abstracts and titles were screened to include only peer-reviewed primary research reports specific for closed head TBI. Given the goal of this systematic review to summarize histopathological evidence of post-traumatic tau accumulation, we included articles for full-text review if they contained any reference to tau protein assessment (including phospho-tau, phosphorylated tau, hyperphosphorylated tau, pTau, p-Tau, pp-Tau, insoluble tau). To avoid

excluding studies that may have assessed tau but not included this in the abstract or title we also conducted a full text search on papers that had any of the following keywords in the title or abstract: amyloid, neurodegeneration, inflammation, astrocyte, microglia, and axonal injury.

After full text review we excluded studies based on following criteria: 1) text not in English; 2) not published in a peer-reviewed journal; 3) review article, 4) conference abstract, 5) analysis done in tau-transgenic mice without data on wild-type mice, 6) no assessment of tau; 7) use of penetrating brain injury models (including controlled cortical impact and fluid percussion injury models). Reference lists were browsed to identify potential additional studies of interest. The final list of included studies was decided on consensus.

Retrieval of information from full-text articles

All articles were imported into EndNote™ (version X9, www.endnote.com). For collection of information on methods of each of these articles, an excel spreadsheet was created. The title and doi were collected as general identifiers. For characterization of mouse models assessing for tau pathology, the following information was collected: injury model used, time of histological tau assessment relative to the first injury, mode of tau assessment, and location of tau pathology in the brain. Finally, we collected histological outcomes that have been associated with CTE, including assessment of axonal and neuronal injury, astrogliosis, microglial activation, TDP-43 pathology, as well as presence of amyloid pathology, α-synuclein, cerebral microbleeds, and evidence of BBB disruption. Information on outcome variables was collected only for wild type animals. If any transgenic animals were used or treatments were reported in the publication, the outcomes observed in transgenic mice and with treatment were not considered as content for this review. No attempt was undertaken to contact authors to obtain additional data.

Supplemental Figures and Legends

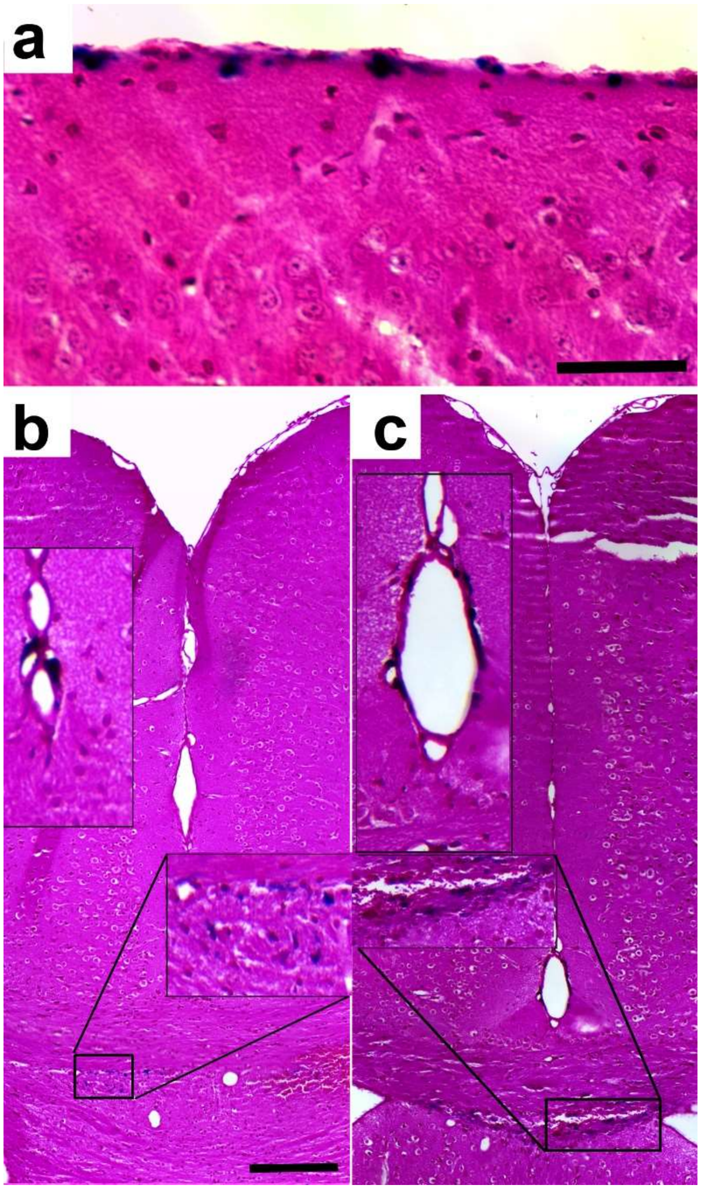

Supplemental Figure 1 Presence of microvascular injury as assessed by Prussian blue staining

Hemosiderin laden macrophages indicative microvascular injury in subpial locations **(a)** and at the depth of the superior longitudinal fissure **(b, c)**. Overall stable in Prussian blue staining from 1 week **(b)** to 4 weeks **(c)** after repetitive traumatic brain injury (rTBI) around vessels (large insets) and at the grey-white matter junction between cortex and corpus callosum (small insets). Scale bars are 40 μm **(in a)** and 250 μm **(in b, c)**.

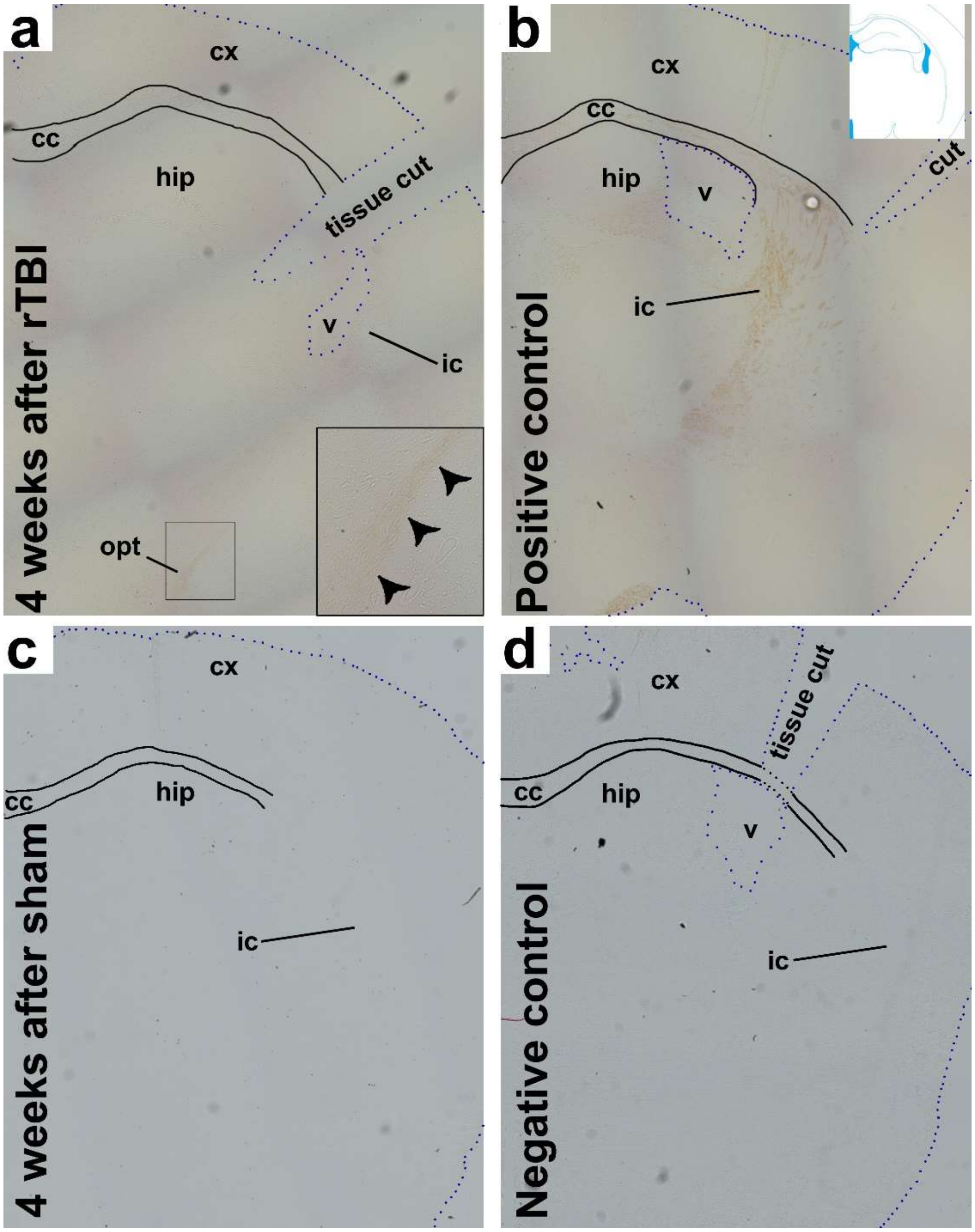

Supplemental Figure 2 Evidence of prior blood brain barrier disruption as assessed by immunoglobulin G (IgG) staining

(**a**) Subtle IgG staining within the optic tract (opt) at 4 weeks after repetitive traumatic brain injury (arrowheads in inset). **(b)** Significant IgG staining extending from the corpus callosum (cc) to the internal capsule (ic) at 4 weeks after a different TBI paradigm serving as positive control. Absent IgG staining in (**c**) sham animals and (**d**) negative control. Cartoon depicts approximate location no of the brain samples. Cx indicates cerebral cortex; cc=corpus callosum, hip=hippocampus, ic=internal capsule, opt=optic tract, v=ventricle, tissue cut=post mortem marker to identify the ipsilateral side. Blue dots outline the tissue section and ventricle.

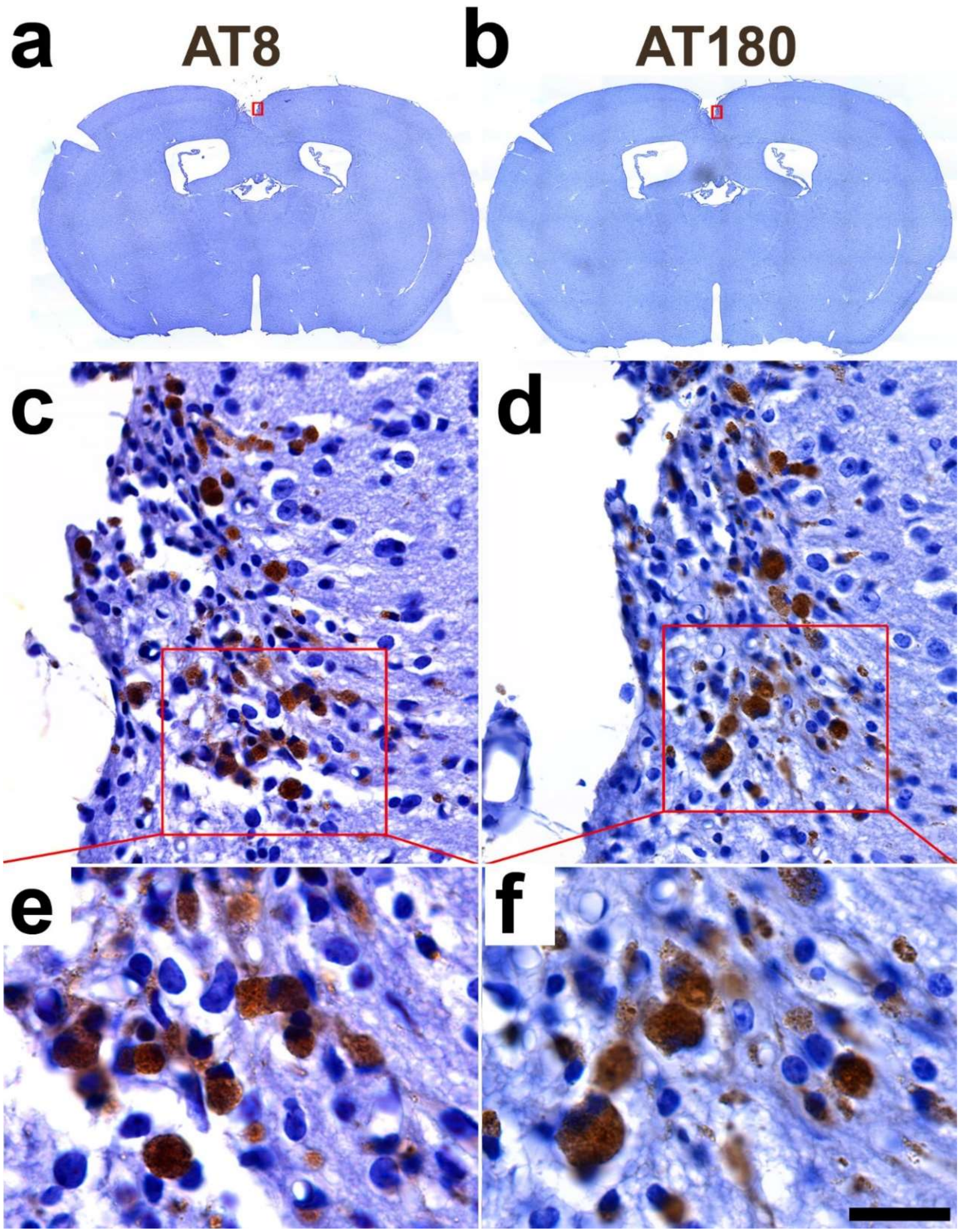

Supplemental Figure 3 Similar specificity of AT8 versus AT180

Immunohistochemistry for AT8 (**a, c, e**) and AT180 (**b, d, f**) depicts overall similar distribution of tau in adjacent sections of mouse brain. Scale bars correspond to 1 mm (in **a-b**) and 30 µm (in **e-f**).

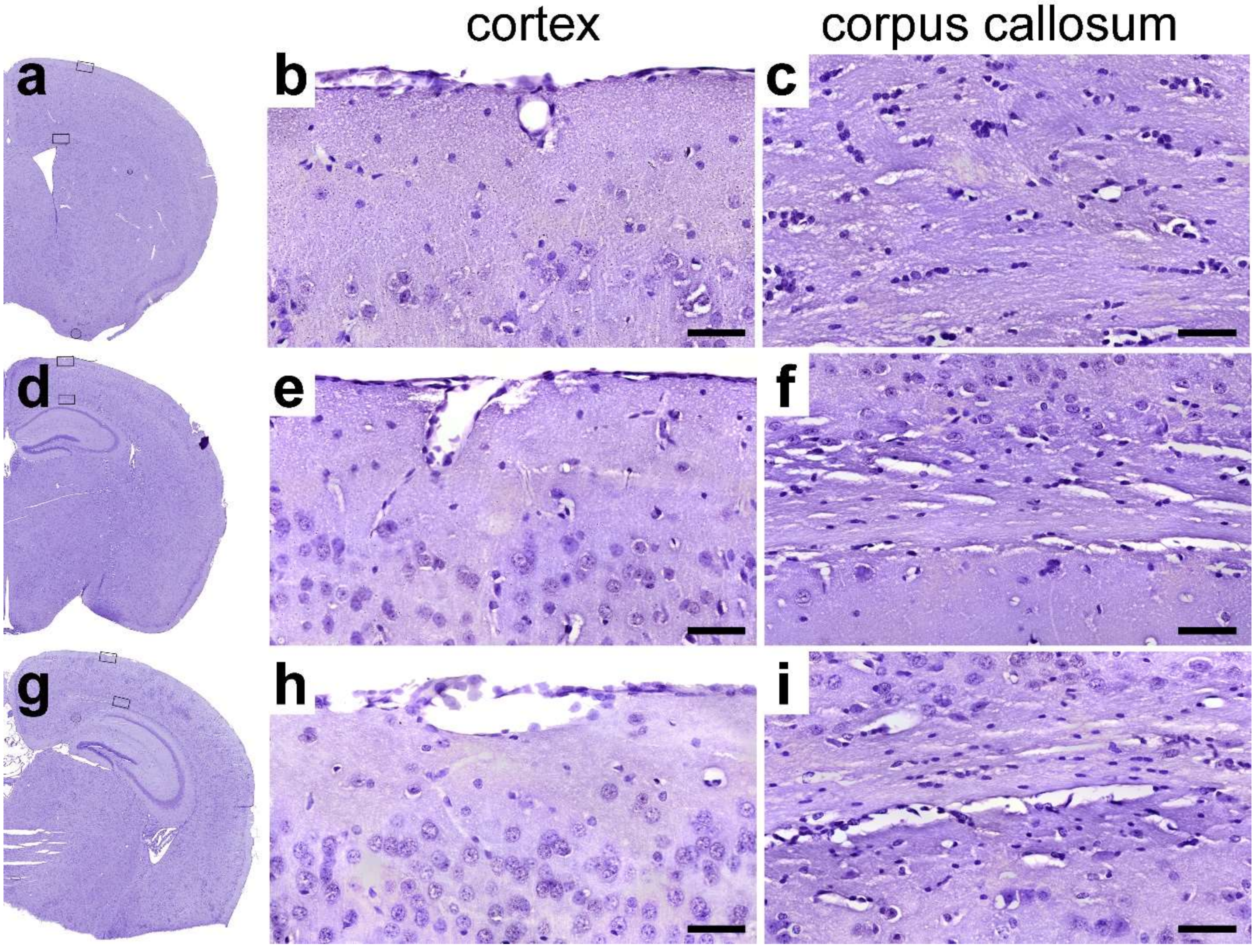

Supplemental Figure 4 Absent p-Tau accumulation in aged sham operated animals.

Absent AT8 staining in sham operated animals in the cerebral cortex (b, e, h) and corpus callosum (c, f, i) at 12 months after surgery. Photomicrographs were taken from the ipsilateral hemisphere at the locations indicated by black boxes in panels (a, d, g). Note, that for presentation purpose, image contrast and brightness of the histological figures was enhanced with Photoshop and by using the same settings across all panels. Scale bars are 30 µm.

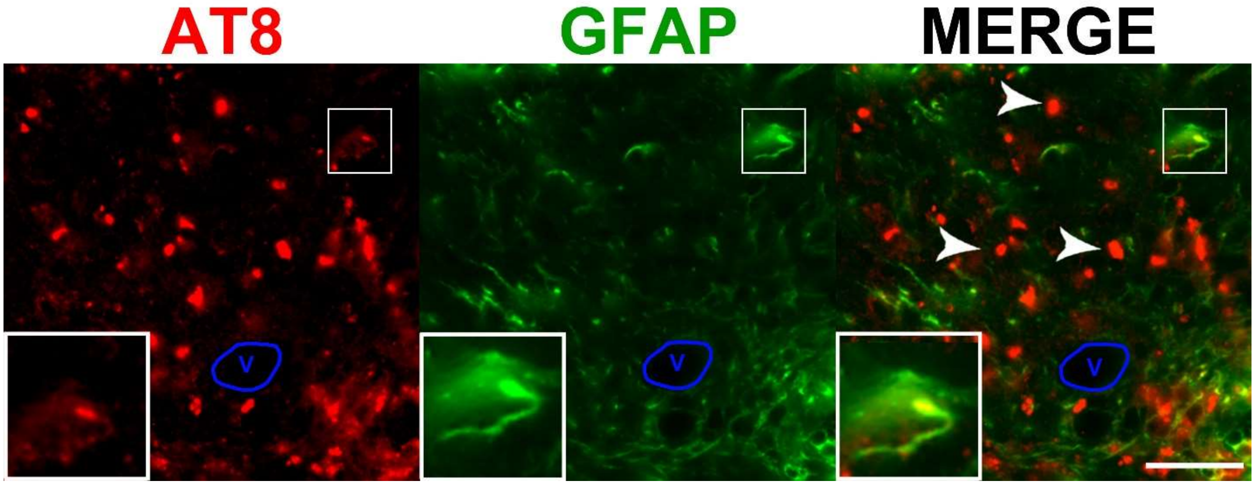

Supplemental Figure 5 Perivascular p-Tau in astrocytes

Double staining indicates colocalization (inset) of hyperphosphorylated tau (p-Tau; AT8) with and astrocyte (GFAP) around a vessel (v) in the ipsilateral cerebral cortex at 4 weeks after rTBI. Examples of AT8 positive cells without GFAP staining (white arrowheads) consistent with tau accumulation in perivascular neurons. Scale bar is 50 µm.

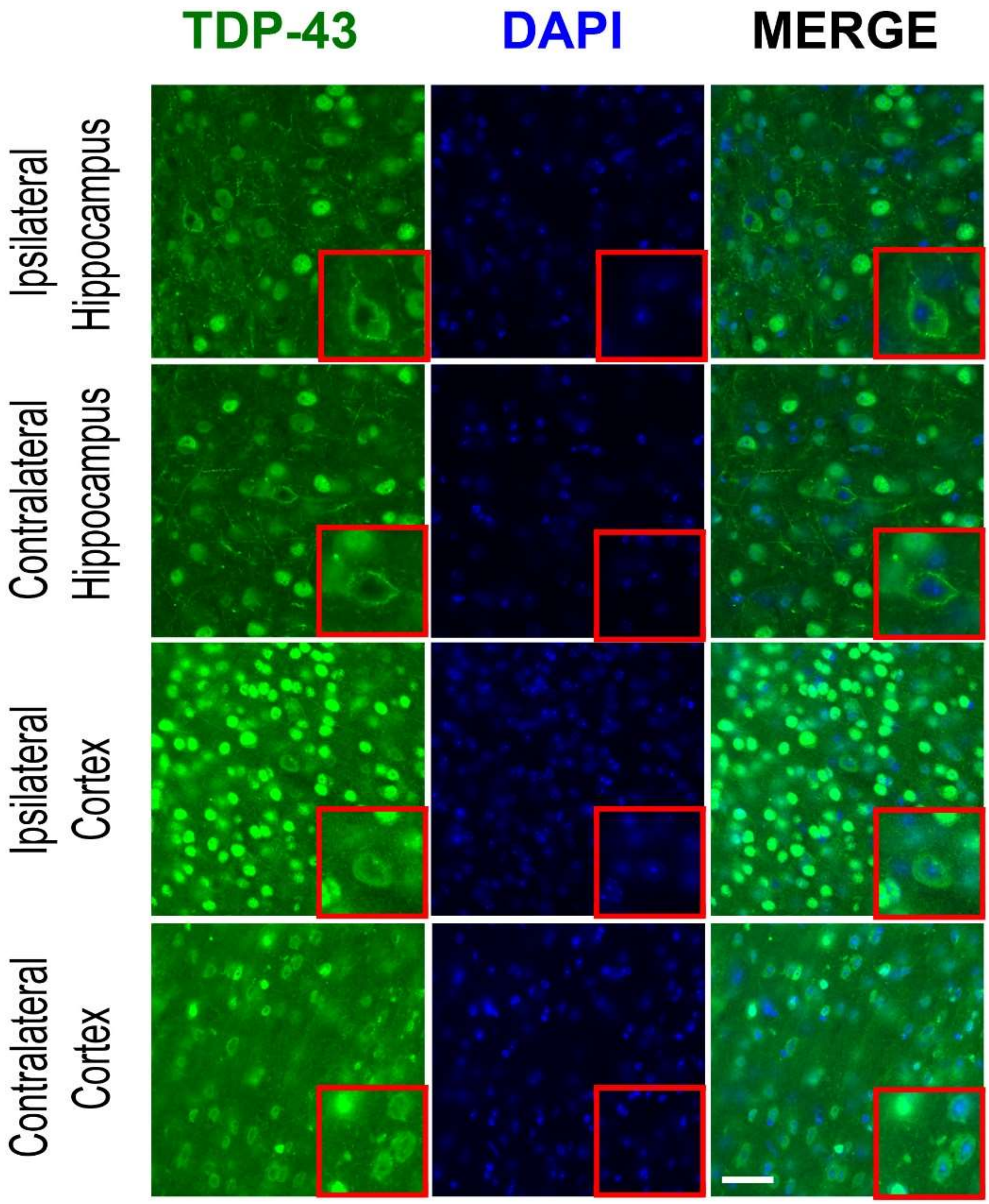

Supplemental Figure 6 Nuclear loss and cytoplasmic localization of TDP-43 in the non-traumatized cerebral cortex and bilateral CA1 of the hippocampus

Examples of nuclear loss and cytoplasmatic localization of TDP-43 at 24 weeks in cortex and hippocampus bilaterally (insets). Scale bar is 50 µm.

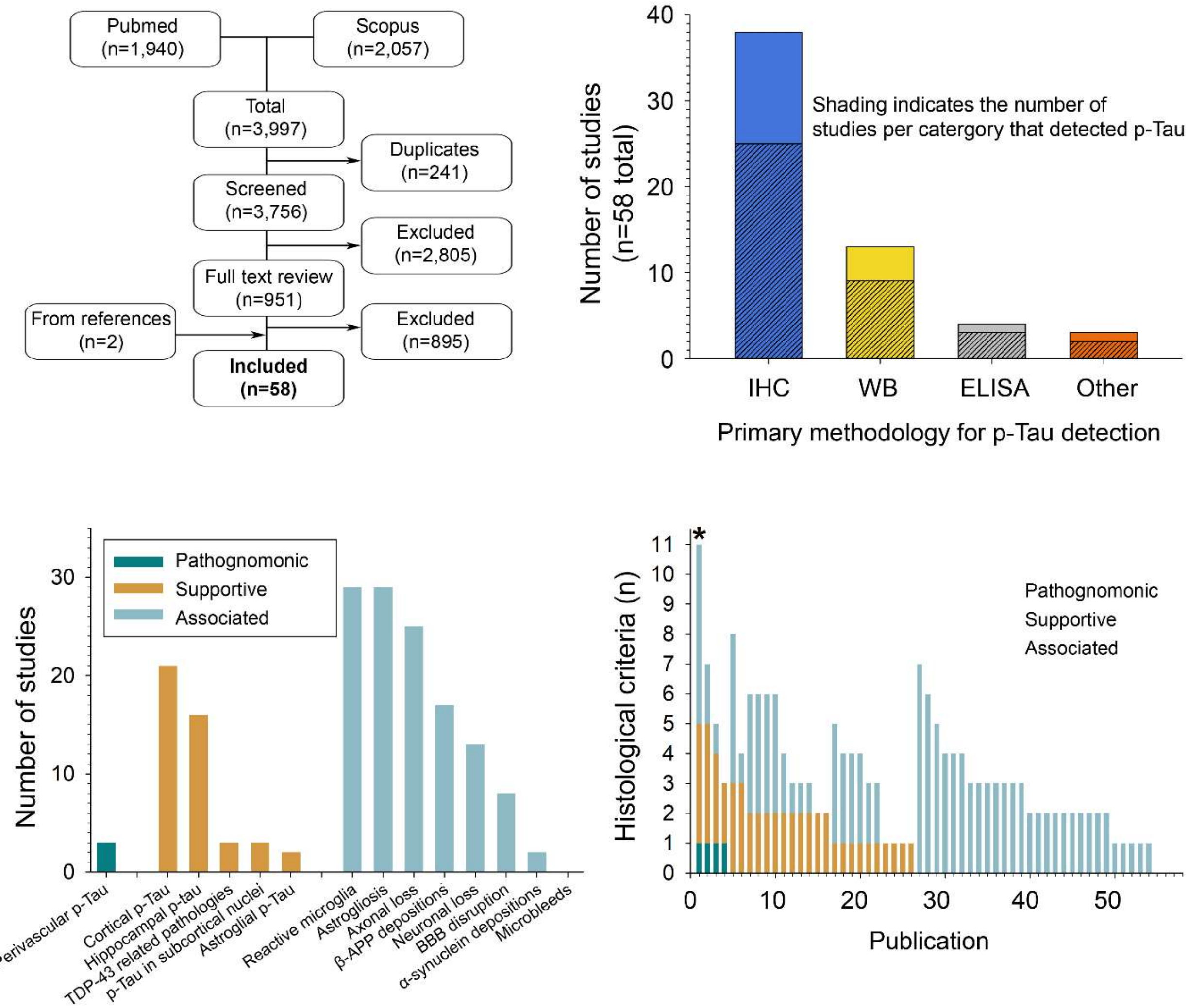

Supplemental Figure 7 Systematic literature review. (a) CONSORT flow diagram. Identification through searches on two separate web-based platforms yielded 3,756 articles. Abstracts were screened with 2,805 articles excluded. After full-text examination of the remaining 951 articles, a total of 58 closed head traumatic brain injury models that assessed tau changes were included in our review. **(b)** Methodologies used to determine the presence of tau pathology. **(c)** Frequency of reported chronic traumatic encephalopathy pathology across all studies and **(d)** within each included study stratified according to pathognomonic, supportive, and associated pathologies. *Denotes the current study. IHC indicates immunohistochemistry; WB, Western blot; ELISA, enzyme-linked immunosorbent assay.

NOTE: Please save this file locally before filling in the table, DO NOT work on the file within your internet browser as changes will not be saved. Adobe Acrobat Reader (available free here) is recommended for completion.

The ARRIVE guidelines 2.0: author checklist

The ARRIVE Essential 10

These items are the basic minimum to include in a manuscript. Without this information, readers and reviewers cannot assess the reliability of the findings.

Item		Recommendation	Section/line number, or reason for not reporting
**Study design**	1	For each experiment, provide brief details of study design including:	
		a. The groups being compared, including control groups. If no control group has been used, the rationale should be stated.	p7
		b. The experimental unit (e.g. a single animal, litter, or cage of animals).	p7
**Sample size**	2	a. Specify the exact number of experimental units allocated to each group, and the total number in each experiment. Also indicate the total number of animals used.	p7
		b. Explain how the sample size was decided. Provide details of any *a priori* sample size calculation, if done.	p7
**Inclusion and exclusion criteria**	3	a. Describe any criteria used for including and excluding animals (or experimental units) during the experiment, and data points during the analysis. Specify if these criteria were established *a priori*. If no criteria were set, state this explicitly.	p7
		b. For each experimental group, report any animals, experimental units or data points not included in the analysis and explain why. If there were no exclusions, state so.	p7
		c. For each analysis, report the exact value of *n* in each experimental group.	p7
**Randomisation**	4	a. State whether randomisation was used to allocate experimental units to control and treatment groups. If done, provide the method used to generate the randomisation sequence.	p7
		b. Describe the strategy used to minimise potential confounders such as the order of treatments and measurements, or animal/cage location. If confounders were not controlled, state this explicitly.	p7
**Blinding**	5	Describe who was aware of the group allocation at the different stages of the experiment (during the allocation, the conduct of the experiment, the outcome assessment, and the data analysis).	p7
**Outcome measures**	6	a. Clearly define all outcome measures assessed (e.g. cell death, molecular markers, or behavioural changes).	p12
		b. For hypothesis-testing studies, specify the primary outcome measure, i.e. the outcome measure that was used to determine the sample size.	
**Statistical methods**	7	a. Provide details of the statistical methods used for each analysis, including software used.	p13
		b. Describe any methods used to assess whether the data met the assumptions of the statistical approach, and what was done if the assumptions were not met.	p13
**Experimental animals**	8	a. Provide species-appropriate details of the animals used, including species, strain and substrain, sex, age or developmental stage, and, if relevant, weight.	p7
		b. Provide further relevant information on the provenance of animals, health/immune status, genetic modification status, genotype, and any previous procedures.	p7
**Experimental procedures**	9	For each experimental group, including controls, describe the procedures in enough detail to allow others to replicate them, including:	pp 7-8
		a. What was done, how it was done and what was used.	pp 7-8
		b. When and how often.	
		c. Where (including detail of any acclimatisation periods).	pp 7-8
		d. Why (provide rationale for procedures).	pp 7-8
**Results**	10	For each experiment conducted, including independent replications, report:	Fig 2-6
		a. Summary/descriptive statistics for each experimental group, with a measure of variability where applicable (e.g. mean and SD, or median and range).	
		b. If applicable, the effect size with a confidence interval.	

The Recommended Set

These items complement the Essential 10 and add important context to the study. Reporting the items in both sets represents best practice.

Item		Recommendation	Section/line number, or reason for not reporting
**Abstract**	11	Provide an accurate summary of the research objectives, animal species, strain and sex, key methods, principal findings, and study conclusions.	pp 3-4
**Background**	12	a. Include sufficient scientific background to understand the rationale and context for the study, and explain the experimental approach.	pp 5-6
		b. Explain how the animal species and model used address the scientific objectives and, where appropriate, the relevance to human biology.	pp 5-6
**Objectives**	13	Clearly describe the research question, research objectives and, where appropriate, specific hypotheses being tested.	p6
**Ethical statement**	14	Provide the name of the ethical review committee or equivalent that has approved the use of animals in this study, and any relevant licence or protocol numbers (if applicable). If ethical approval was not sought or granted, provide a justification.	p36
**Housing and husbandry**	15	Provide details of housing and husbandry conditions, including any environmental enrichment.	p7
**Animal care and monitoring**	16	a. Describe any interventions or steps taken in the experimental protocols to reduce pain, suffering and distress.	pp 7-8
		b. Report any expected or unexpected adverse events.	pp 7-8
		c. Describe the humane endpoints established for the study, the signs that were monitored and the frequency of monitoring. If the study did not have humane endpoints, state this.	pp 7-8
**Interpretation/ scientific implications**	17	a. Interpret the results, taking into account the study objectives and hypotheses, current theory and other relevant studies in the literature.	pp 21-26
		b. Comment on the study limitations including potential sources of bias, limitations of the animal model, and imprecision associated with the results.	
**Generalisability/ translation**	18	Comment on whether, and how, the findings of this study are likely to generalise to other species or experimental conditions, including any relevance to human biology (where appropriate).	pp 21-26
**Protocol registration**	19	Provide a statement indicating whether a protocol (including the research question, key design features, and analysis plan) was prepared before the study, and if and where this protocol was registered.	na
**Data access**	20	Provide a statement describing if and where study data are available.	p36
**Declaration of interests**	21	a. Declare any potential conflicts of interest, including financial and non-financial. If none exist, this should be stated.	p36
		b. List all funding sources (including grant identifier) and the role of the funder(s) in the design, analysis and reporting of the study.	p36

PRISMA-S Checklist

Section/topic	#	Checklist item	Location(s) Reported
**INFORMATION SOURCES AND METHODS**			
Database name	1	Name each individual database searched, stating the platform for each.	Pp 13-14
Multi-database searching	2	If databases were searched simultaneously on a single platform, state the name of the platform, listing all of the databases searched.	Suppl. p. 3
Study registries	3	List any study registries searched.	na
Online resources and browsing	4	Describe any online or print source purposefully searched or browsed (e.g., tables of contents, print conference proceedings, web sites), and how this was done.	Suppl pp. 3-4
Citation searching	5	Indicate whether cited references or citing references were examined, and describe any methods used for locating cited/citing references (e.g., browsing reference lists, using a citation index, setting up email alerts for references citing included studies).	Suppl p. 4
Contacts	6	Indicate whether additional studies or data were sought by contacting authors, experts, manufacturers, or others.	Suppl p. 4
Other methods	7	Describe any additional information sources or search methods used.	na
**SEARCH STRATEGIES**			
Full search strategies	8	Include the search strategies for each database and information source, copied and pasted exactly as run.	Suppl p. 3
Limits and restrictions	9	Specify that no limits were used, or describe any limits or restrictions applied to a search (e.g., date or time period, language, study design) and provide justification for their use.	Suppl p. 3
Search filters	10	Indicate whether published search filters were used (as originally designed or modified), and if so, cite the filter(s) used.	Suppl p. 3
Prior work	11	Indicate when search strategies from other literature reviews were adapted or reused for a substantive part or all of the search, citing the previous review(s).	na
Updates	12	Report the methods used to update the search(es) (e.g., rerunning searches, email alerts).	na

Dates of searches	13	For each search strategy, provide the date when the last search occurred.	Suppl p. 3
**PEER REVIEW**			
Peer review	14	Describe any search peer review process.	na
**MANAGING RECORDS**			
Total Records	15	Document the total number of records identified from each database and other information sources.	pp 13-14
Deduplication	16	Describe the processes and any software used to deduplicate records from multiple database searches and other information sources.	pp 13-14<br>Suppl. p. 4

PRISMA-S: An Extension to the PRISMA Statement for Reporting Literature Searches in Systematic Reviews
Rethlefsen ML, Kirtley S, Waffenschmidt S, Ayala AP, Moher D, Page MJ, Koffel JB, PRISMA-S Group.
Last updated February 27, 2020.
